# Supplementary material for: A phase I/II study of triple-mutated oncolytic herpes virus G47∆ in patients with progressive glioblastoma
Source: Nat Commun. 2022 Jul 21;13:4119. doi: 10.1038/s41467-022-31262-y (PMC9304402; doi:10.1038/s41467-022-31262-y)
Supplement: Supplementary file 1 — Supplementary Information [file 41467_2022_31262_MOESM1_ESM.pdf]

Supplementary Information for

A Phase I/II study of triple-mutated oncolytic herpes virus G47Δ in patients with progressive glioblastoma

Tomoki Todo, M.D., Ph.D.[1,2] Yasushi Ino, M.D., Ph.D.[1,2] Hiroshi Ohtsu, M.Sc.,[3,4] Junji Shibahara, M.D., Ph.D.,[5] Minoru Tanaka, M.D., Ph.D.[1,2]

1. *Division of Innovative Cancer Therapy, Advanced Clinical Research Center, and Department of Surgical Neuro-Oncology, The Institute of Medical Science, The University of Tokyo, Tokyo, Japan*
2. *Department of Neurosurgery, The University of Tokyo Hospital, Tokyo, Japan*
3. *Department of Data Science, National Center for Global Health and Medicine in Japan, Tokyo, Japan*
4. *Leading Center for the Development and Research of Cancer Medicine, Juntendo University, Tokyo, Japan.*
5. *Department of Pathology, Kyorin University School of Medicine, Tokyo, Japan*

This pdf file contains:

Supplementary Figure 1. Original dose escalation scheme.

Supplementary Figure 2. MR images of all patients comparing pre- and post-administration and of target sites.

Supplementary Figure 3. Time course of cross-sectional area of tumor for individual patients.

Supplementary Table 1. Inclusion and exclusion criteria

Supplementary Table 2. Cumulative adverse events

Supplementary Table 3. Viral shedding

Supplementary Table 4. Peripheral blood counts at baseline and at days 30 and 60 after treatment

Supplementary Table 5. Details of primary antibody and antigen retrieval system used in immunohistochemistry

Supplementary Note 1. Clinical study protocol (redacted and translated)

Supplementary Figure 1. Original dose escalation scheme.

The original dose escalation scheme of the phase I part was as follows: If Grade ≥3 adverse events attributable to G47Δ were absent in a cohort, the study proceeded to the next cohort. However, if such adverse events were observed in one patient, 3 additional patients were included, and the study proceeded to the following cohort provided that no such adverse events were observed in the additional 3 patients. The study was to be terminated if 2 or more patients in Cohort 1 (3.0×10^8 pfu/dose) had Grade ≥3 adverse events attributable to G47Δ. If 2 or more patients in Cohort 2 or higher experienced such adverse events, a single lower dose was considered as the tentative maximum tolerated dose (MTD). A total of 6 additional patients were to be enrolled in the tentative MTD cohort. If no more than 1 patient had a Grade ≥3 adverse event attributable to G47Δ, this dose was considered the MTD. If 2 or more patients experienced G47Δ-related Grade ≥3 adverse events, a further one-level lower dose was considered as the tentative MTD, and up to 6 additional patients were enrolled at that dose. After the set dose or MTD was determined, a further 12 patients were enrolled to be treated in the phase II part at that dose. If at least 4 of the 12 patients experienced G47Δ-related Grade ≥3 adverse events during treatment at that dose, the study was discontinued.

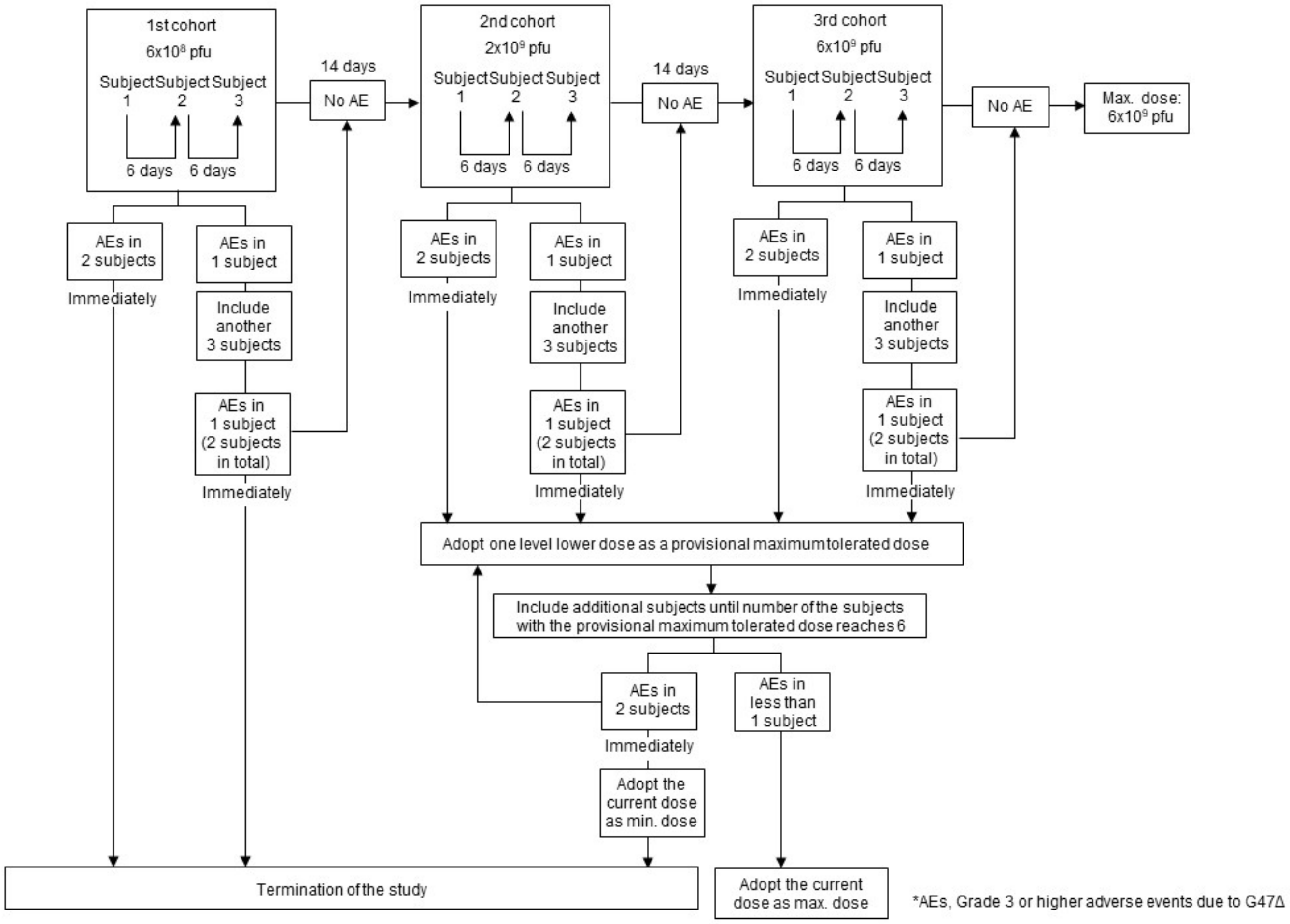

Supplementary Figure 2. MR images of all patients comparing pre- and post-administration and of target sites.

Each target site was the intersection point where the red lines crossed. For each patient, one of two target sites is demonstrated. Post-administration MRI showed enlargement of the entire contrast enhanced lesion and clearing of contrast enhancement at the site of G47Δ injection (arrow), depicting an explosion crater-like appearance for most patients.

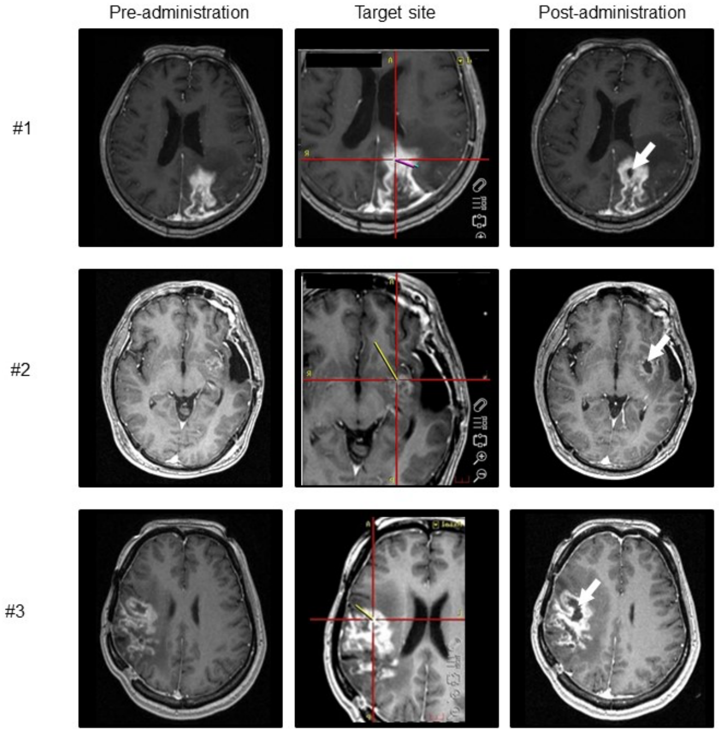

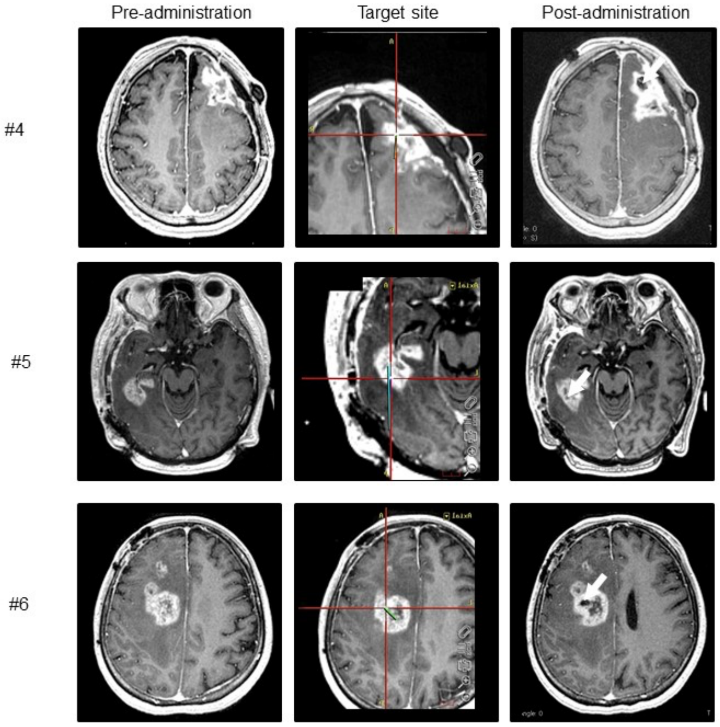

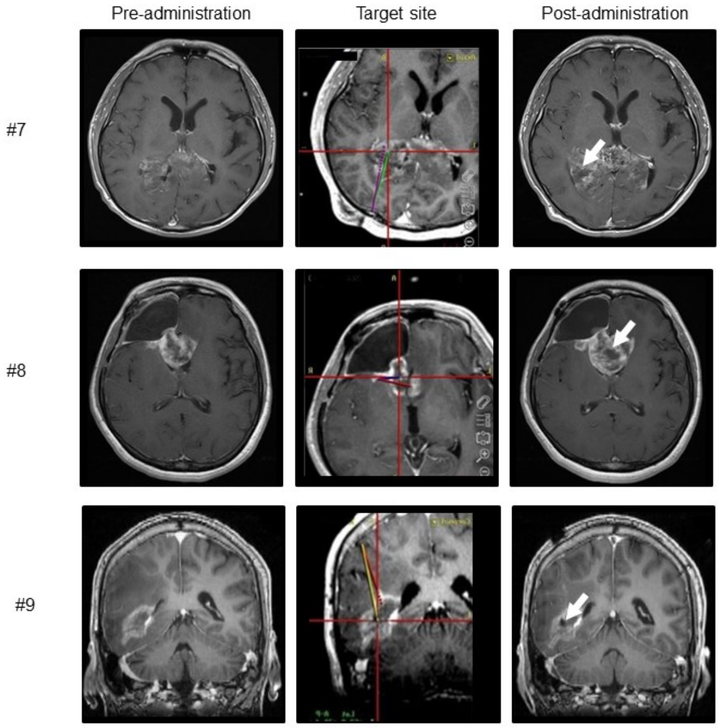
Pre-administration
Target site
Post-administration
#7
#8
#9

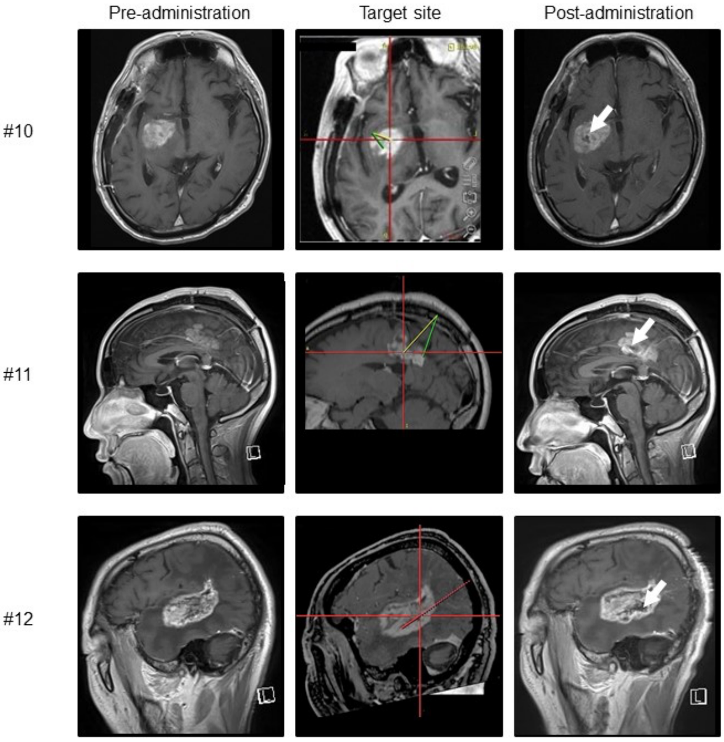
Pre-administration
Target site
Post-administration
#10
#11
#12

#13

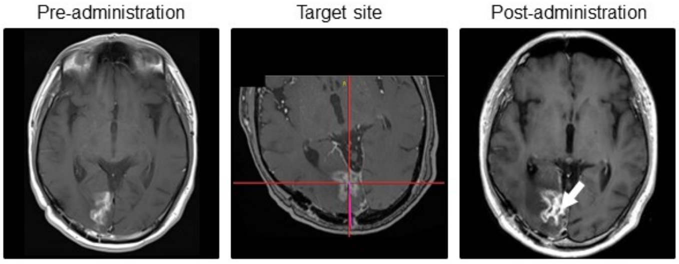

Supplementary Figure 3. Time course of cross-sectional area of tumor for individual patients.

(A) Cross-sectional area of tumors calculated from MRI. Most tumors showed an initial increase in size at day 7, reflecting immunoprogression. (B) Cross-sectional area of tumors depicted as the rate of tumor growth compared with pre-G47Δ administration. One patient (#3) showed shrinkage of the tumor 1 month after G47Δ administration compared with pre-administration, although the response was judged as SD according to WHO response criteria. *, IDH1 mutated.
Source data are provided as a Source Data file.

A

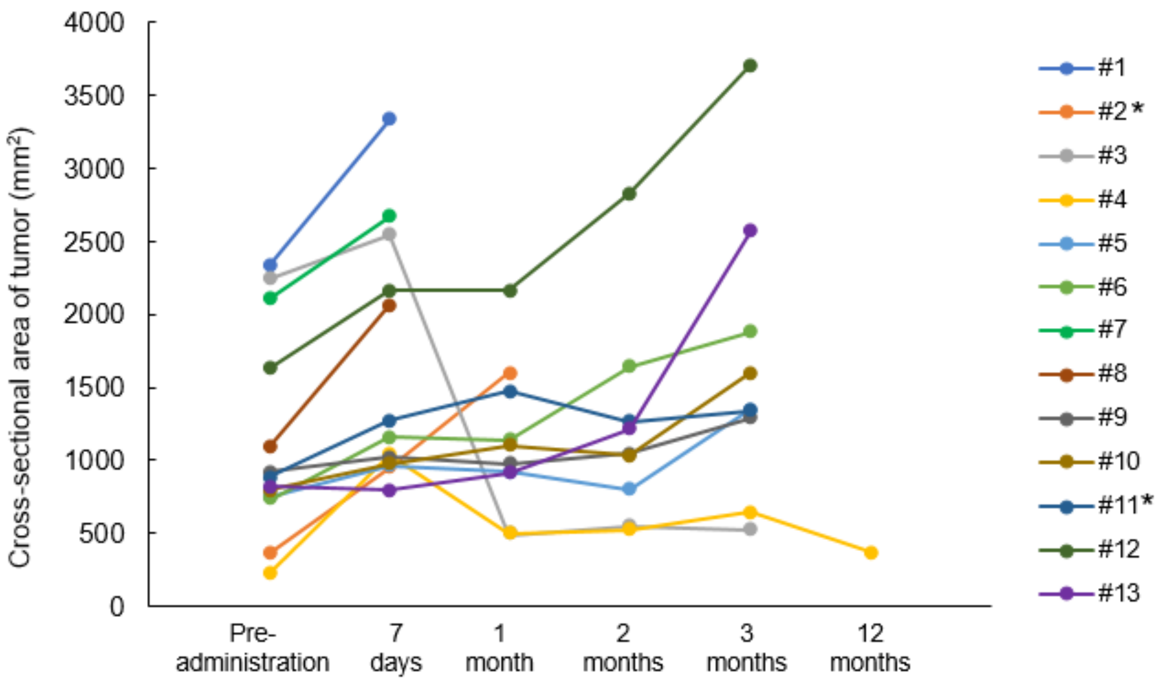

B

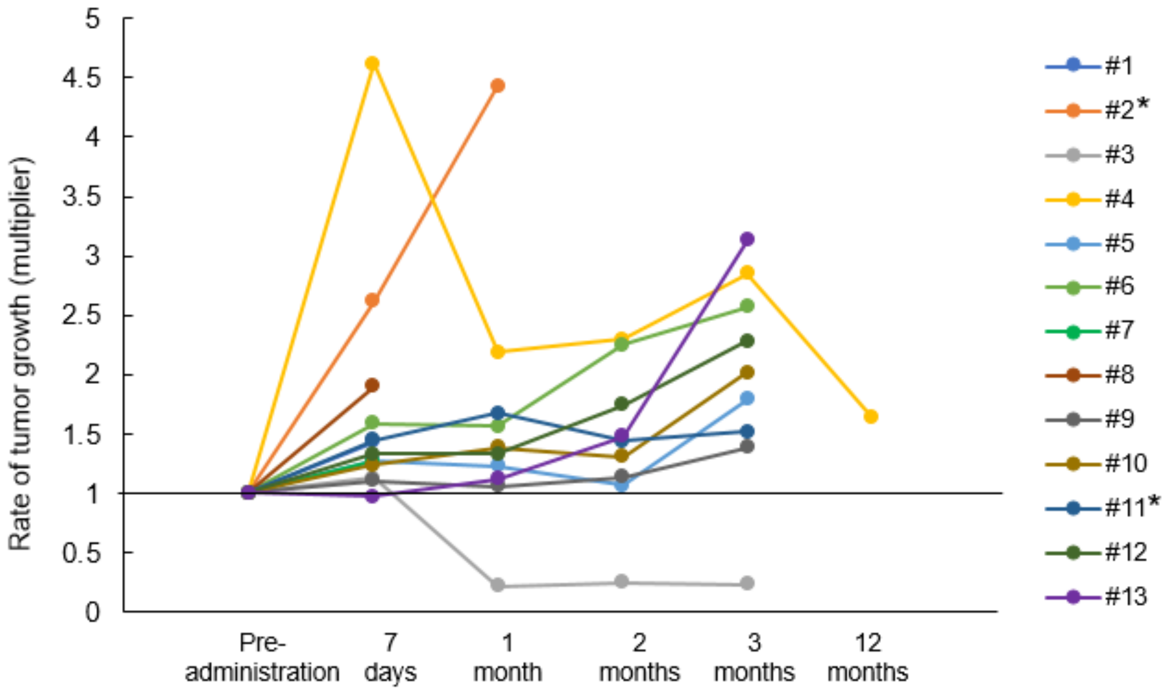

Supplementary Table 1. Inclusion and exclusion criteria

Inclusion criteria

Included patients met all of the following criteria:

- Patients who have undergone surgery (stereotactic biopsy or craniotomy) at the time of initial or recurrent disease and have been pathologically diagnosed with glioblastoma and have not responded to radiotherapy for at least 30 days after surgery
- Patients in whom the site of the tumor does not meet the following exclusion criteria
- Patients with lesions enhancing at least 1.0 cm on MRI within 14 days prior to G47Δ administration
- With or without prior chemotherapy
- Karnofsky Performance Scale (KPS) in >60% or KPS due to hemiplegia caused by surgical removal of tumors in 50% of patients
- Patients aged 18 years or older at the time of informed consent
- Patients who are allowed to receive steroids but at a fixed dose within 1 week before G47Δ administration
- Patients willing to perform barrier-type contraception for at least 6 months after G47Δ administration
- Patients who are likely to survive longer than a month
- Patients with no abnormalities that met the exclusion criteria and normal function of major organs in various examinations
- Patients who are capable and willing to give written informed consent

Exclusion criteria

Patients who met any of the following criteria were excluded:

Medical history

- History or coexistence of other cancers, excluding curable in situ cancer of the cervix and basal cell carcinoma or squamous cell carcinoma of the skin
- History of encephalitis, multiple sclerosis, or other CNS infections
- Positive or previous HIV
- History or coexistence of alcohol or other drug poisoning
- When MRI examination (using contrast media) is contraindicated. e.g., pacemakers, duration-infusion pumps placed in the body, MRI contrast allergies
- Other medical or psychiatric abnormalities that may make it difficult to adhere to protocol treatment

Tumor location

- Presence of extracerebral metastatic disease
- Presence of multiple (two or more) glioblastoma lesions in the skull
- Ventricles, brainstem, or posterior fossa, or tumors that must be reached via the ventricles
- Presence of subependymal and subarachnoid dissemination

Laboratory evidence

- Leukocytes $\leq 2.0 \times 10^3$ per mm^3, neutrophils $\leq 1.0 \times 10^3$ per mm^3, platelets ≤100,000 per mm^3, haemoglobin (Hb) ≤9.0 g/dL, INR or activated partial thromboplastin time (APTT) > 1.3 times the institutional standard

- Serum creatinine ≥1.7 mg/dL
- Hepatic transaminases (AST or ALT) > 4 times the reference level of the trial site
- Total bilirubin or direct bilirubin > 1.5 mg/dL

Comorbidity

- Presence of active herpesvirus infection
- At study entry, antiviral (acyclovir, valacyclovir) treatment for HSV is required
- Presence of an active, uncontrolled infection that are off-label for surgery
- Poor control or severe heart failure, diabetes mellitus, hypertension, interstitial pneumonia, renal failure, autoimmune disease, etc.

Allergy to relevant drugs

- Presence of allergy to anti-HSV drug (acyclovir)

Concomitant medication/treatment

- Administration of other clinical study drugs within 30 days before administration of G47Δ
- Immunotherapy (interferon, etc.) given within 6 weeks before G47Δ administration
- Administration of vaccine within 30 days before administration of G47Δ
- Brain tumor resection within 30 days before G47Δ administration
- History of gene therapy or viral therapy other than G47Δ
- History or registered person of G47Δ virus therapy

Pregnancy or lactation

- Pregnancy or breast-feeding women

Other

- Other than the above, when deemed inappropriate by the physician

Supplementary Table 2. Cumulative adverse events

#	Event	Grade CTCAE v3.0	Relationship to G47Δ	Days post-G47Δ
1	Vomiting	2	5	0
	Hypotension	1	5	5
	Hemorrhage with surgery	1	3	5
	Elevated GGT	1	4	7
	Headache	1	4	8
	Elevated ALT, SGPT	1	4	8
	Seizure	2	5	10
	Vomiting	1	5	0
	Headache	1	4	0
	Lymphopenia	4	5	1
	Anorexia	1	5	5
2	Vomiting	2	3	1
	Headache	1	3	0
	Fever	1	3	1
	Neuropathy: lateral deviation of eye	1	3	1
	Vomiting	2	3	0
	Headache	1	3	0
	Fever	1	3	1
3	Nausea	1	3	0
	Headache	1	3	0
	Fever	1	3	1
	Desquamation	1	5	6
	Headache	1	3	0
	Vomiting	1	4	23
	Flu-like syndrome	1	5	40
4	Nausea	1	5	0
	Fever	1	3	1
	Seizure	2	3	4
	Pruritus	1	5	4
	Seizure	2	3	0
	Fever	1	3	0
	Decreased leukocytes	2	4	8
5	Wound pain	1	1	0
	Seizure	2	2	4
	Seizure	2	2	9
	Wound pain	1	1	0
	Hypertension	2	4	0
	Fever	1	4	2
6	Wound pain	1	1	0
	Seizure	2	3	0
	Decreased hemoglobin	1	3	1
	Pruritus	1	5	4
	Hemorrhage: Genitourinary (Urethra)	1	5	5
	Wound pain	1	1	0

	Seizure	2	2	0
	Hemorrhage with surgery	1	1	0
7	Headache	1	2	0
	Fever	2	2	0
	Decreased leukocytes	3	3	4
	Vomiting	1	2	5
	Tremor	1	3	5
	Headache	1	2	5
	Fever	2	2	2
	Anorexia	1	3	4
	Nausea	2	3	5
	Headache	1	3	5
	Headache	1	4	15
	Headache	3	4	22
8	Vomiting	1	3	0
	Headache	1	1	0
	Fever	1	3	1
	Lymphopenia	2	4	1
	Lymphopenia	2	4	12
	Vomiting	3	3	0
	Headache	1	3	0
	Fever	1	3	0
	Hemorrhage with surgery	1	1	1
9	—	—	—	—
10	Fever	1	3	1
	Fever	1	3	3
11	Sinus bradycardia	2	5	0
	Elevated ALT, SGPT	1	4	1
	Neuropathy: motor	3	4	1
	Decreased leukocytes	1	4	6
	Headache	1	5	0
	Elevated INR	1	4	1
	Fall	1	5	2
	Decreased leukocytes	1	4	4
	Fall	1	5	5
	Pain: buttock	2	5	5
	Neuropathy: sensory	2	5	32
	Headache	2	5	60
	Neuropathy: motor (pharynx, palate, sternomastoid)	3	5	60
	Fatigue	2	5	95
12	Fever	1	2	1
	Anorexia	1	3	1
	Nausea	1	3	1
	Headache	1	4	1
	Decreased hemoglobin	1	4	6
	Hyperbilirubinemia	1	4	1
	Vomiting	2	2	2
	Constipation	1	4	40
	Rhinitis	1	4	40
13	Decreased leukocytes	2	3	5
	Headache	1	5	90

#, patient number.

Relevance: 1: Clearly related 2: Likely related 3: Possibly related 4: Not likely to be related 5: Not related

Days post-G47Δ: days after the first G47Δ administration.

Abbreviations: ALT, alanine aminotransferase; GGT, γ-Glutamyl transpeptidase; INR, (International Normalized Ratio of prothrombin time; SGPT, serum glutamic pyruvic transaminase

Supplementary Table 3. Viral shedding

#	G47Δ DNA (qPCR)			Culture	G47Δ DNA (qPCR)			Culture	G47Δ DNA (qPCR)			Culture
	**Saliva**	**Blood**	**Urine**	**Blood**	**Saliva**	**Blood**	**Urine**	**Blood**	**Saliva**	**Blood**	**Urine**	**Blood**
	**1 day after the first dose**				**1 day after the second dose**				**7 days after the second dose**			
1	N	N	N	N	N	N	N	N	N	N	N	N
2	N	N	N	N	N	N	N	N	N	N	N	N
3	N	N	N	N	N	N	N	N	N	N	N	N
4	N	N	N	N	N	N	N	N	N	N	N	N
5	N	N	N	N	N	N	N	N	N	N	N	N
6	N	N	N	N	N	N	N	N	N	N	N	N
7	N	N	N	N	N	N	N	N	N	N	N	N
8	N	N	N	N	N	N	N	N	N	N	N	N
9	N	N	N	N	N	N	N	N	N	N	N	N
10	N	N	N	N	N	N	N	N	N	N	N	N
11	N	N	N	N	N	N	N	N	N	N	N	N
12	N	N	N	N	N	N	N	N	N	N	N	N
13	N	N	N	N	N	N	N	N	N	N	N	N

#, patient number. N denotes negative.
Abbreviation: qPCR, quantitative polymerase chain reaction

Supplementary Table 4. Peripheral blood counts at baseline and at days 30 and 60 after treatment

#	Baseline				Day 30				Day 60			
	**WBC**	**CD4**	**CD8**	**CD4/CD8**	**WBC**	**CD4**	**CD8**	**CD4/CD8**	**WBC**	**CD4**	**CD8**	**CD4/CD8**
1	3000	172	148	1.16	ND	ND	ND	ND	ND	ND	ND	ND
2	4200	264	253	1.04	4100	240	240	1.00	ND	ND	ND	ND
3	2500	259	210	1.23	3000	250	260	0.96	4900	352	448	0.79
4	3900	336	112	3.00	3600	376	144	2.61	3300	408	157	2.59
5	3500	451	251	1.80	5600	324	229	1.41	3000	291	144	2.01
6	4600	218	307	0.71	4300	149	196	0.76	3100	136	146	0.93
7	2600	132	98	1.34	6300	44	54	0.80	ND	ND	ND	ND
8	9700	796	433	1.84	5700	547	301	1.81	ND	ND	ND	ND
9	7900	263	393	0.67	6400	203	366	0.55	5400	262	604	0.43
10	5500	477	302	1.58	6100	548	285	1.93	3800	330	159	2.08
11	3540	313	339	0.92	3860	303	423	0.72	4080	208	215	1.00
12	3400	595	404	1.47	4140	566	575	0.98	3600	525	435	1.21
13	2760	176	376	0.47	5240	261	721	0.4	4330	252	763	0.33

#, patient number. ND denotes no data.

Abbreviation: CD4, cluster of differentiation 4; CD8, cluster of differentiation 8; WBC, white blood cell

Supplementary Table 5. Details of primary antibody and antigen retrieval system used in immunohistochemistry

Primary antibody	Host	Clone	Maker	Cat. No.	Dilution	Antigen retrieval	Lot No.
CD4	rabbit	EPR6855	Abcam	ab133616	1:250	Citrate buffer pH6	GR3276764-5
CD8	rabbit	SP16	Abcam	ab101500	1:100	Citrate buffer pH6	9116S1711F
FOXP3	rabbit	SP97	Abcam	ab99963	1:50	EDTA buffer pH9	GR3281127-15
HSV	rabbit	polyclonal	Gene Tex	GTX73373	ready-to-use	None	822100458
PD-1/CD279	rabbit	N/A	Spring	E18660	1:50	EDTA buffer pH9	141008LVF
PD-L1	rabbit	E1L3N	Cell Signaling	#13684	1:50	EDTA buffer pH9	0007
p53	mouse	DO-7	Agilent	NCL-L-p53-DO7	1:100	EDTA buffer pH9	6069731
Ki-67	mouse	MIB-1	Dako	M7240	1:200	Citrate buffer pH6	20073124
IDH1 R132H	mouse	H09	Dianova	DIA-H09	1:100	EDTA buffer pH9	211129/02
MGMT	mouse	MT3.1	Abcam	ab39253	1:100	Citrate buffer pH6	GR3422909-2

Antigen retrieval solution

1. Citrate buffer pH6

Antigen Unmasking Solution, Citric Acid Based	VECTOR	H-3300	1:100	ZH0603

2. EDTA buffer pH9

Antigen Unmasking Solution, Tris Based	VECTOR	H-3301	1:100	ZH0610

Secondary antibody: Labelled Polymer method

EnVision+ Dual Link System-HRP	Dako	K4061	11347917

Visualization of antigen-antibody complex

ImmPACT DAB Substrate, Peroxidase (HRP)	VECTOR	SK-4105	ZH1111A

Negative controls were used PBS instead of the primary antibodies.

Supplementary Note 1

Protocol for the Clinical Research of Gene Therapy

Department of Surgical Neuro-oncology, the Institute of Medical Science, University of Tokyo (IMSUT) Hospital

Version 1: Date of Report: 16 Jan 2013

TITLE OF THE CLINICAL RESEARCH OF GENE THERAPY

Clinical Research of Gene Therapy (Oncolytic Virus Therapy) Using Replication-competent Recombinant Herpes Simplex Virus G47delta in Patients with Progressive Glioblastoma

1. NAME AND RESPONSIBILITIES OF THE RESEARCH DIRECTOR AND OTHER INVESTIGATORS IN THE CLINICAL RESEARCH OF GENE THERAPY

(1) Name of the research director

Tomoki Todo	Professor, Division of Innovative Cancer Therapy (Department of Surgical Neuro-oncology), Advanced Clinical Research Center, Institute of Medical Science, the University of Tokyo

Supervision of the clinical research of gene therapy conducted at the Institute of Medical Science, University of Tokyo (IMSUT) Hospital

(2) Name and responsibilities of other investigators than the research director

[Omitted]

2. NAME AND ADDRESS OF THE STUDY SITE

Name: Institute of Medical Science, University of Tokyo (IMSUT) Hospital

Address: 4-6-1 Shirokanedai, Minato-ku, Tokyo 108-8639, Japan

3. OBJECTIVE OF THE CLINICAL RESEARCH OF GENE THERAPY

In this research, recombinant herpes simplex virus type 1 (HSV-1) G47delta[1] will be intratumorally administered in a stereotactic manner to patients with recurrent or progressive glioblastoma despite initial radiation therapy. The dose of G47delta will be increased in three levels per cohort in an open label manner with the primary objective of evaluating the safety, or the types and incidence of adverse events (AEs). The secondary objective is to evaluate the efficacy of G47delta based on the radiographic objective tumor response, overall survival, and progression-free survival.

4. TARGET DISEASE OF THE CLINICAL RESEARCH OF GENE THERAPY AND THE REASON FOR SELECTION

[Omitted]

5. TYPE OF THE GENE AND THE GENE TRANSDUCTION METHOD

[Omitted]

6. SAFETY EVALUATION

[Omitted]

7. REASON FOR CONSIDERING THAT THE CLINICAL RESEARCH OF GENE THERAPY IS FEASIBLE

[Omitted]

8. PROTOCOL FOR THE CLINICAL RESEARCH OF GENE THERAPY

(1) Overall treatment plan including the clinical research of gene therapy

This is an open-label dose escalation study.

[1] Scheme

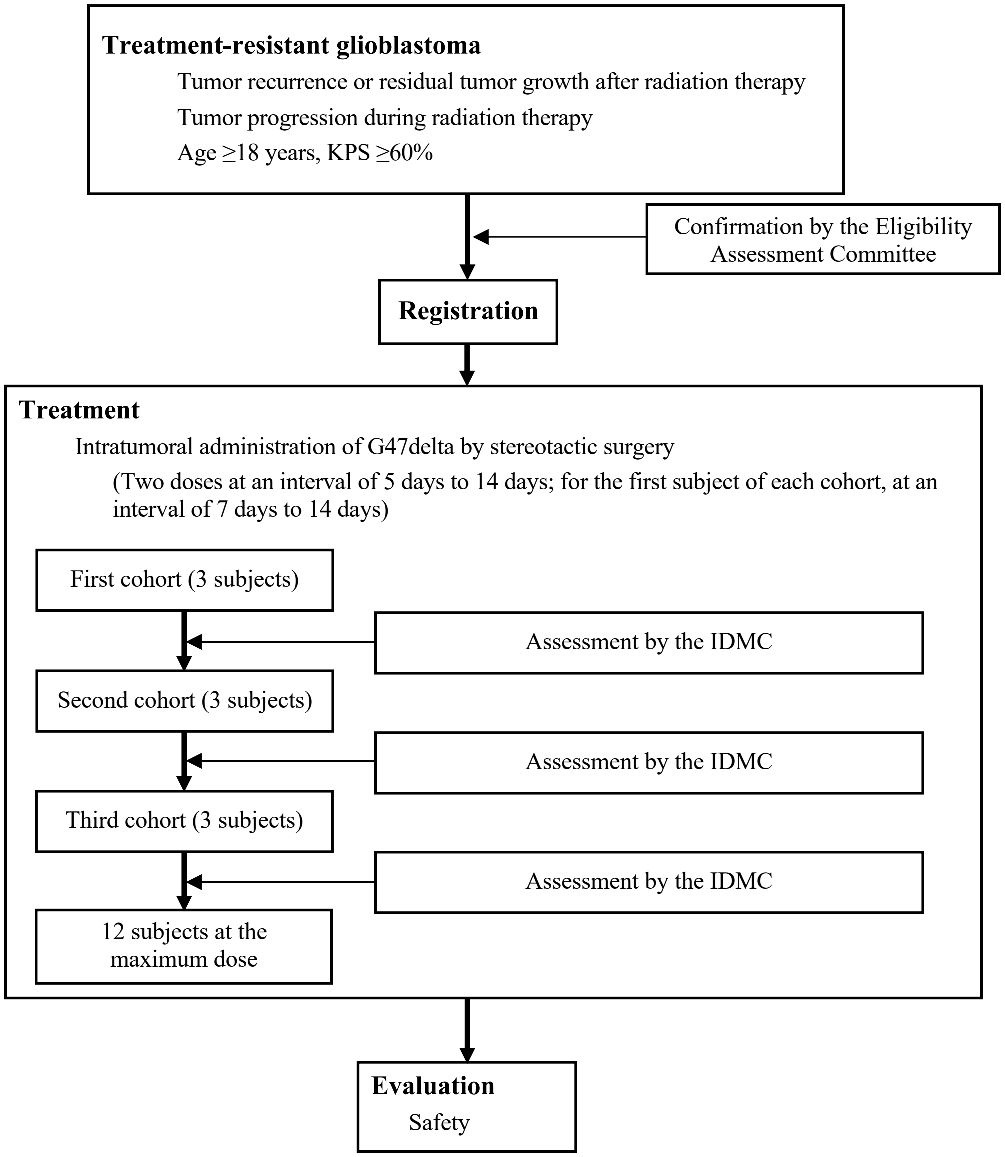

Dose escalation method

Dose Level	Dose (pfu)	Total Dose (pfu)	No. of Subjects
1	3×10^8 pfu	6×10^8 pfu	3
2	1×10^9 pfu	2×10^9 pfu	3
3	3×10^9 pfu	6×10^9 pfu	3

pfu = plaque-forming units

Following stepwise dose escalation, 12 additional subjects will be studied at Dose Level 3 or the maximum tolerated dose.

[2] Target disease and disease stage

The target population of the study consists of patients visiting the IMSUT Hospital (including referred patients) who have recurrent or progressive glioblastoma despite initial radiation therapy, wish to participate in this study, and meet all of the inclusion criteria and none of the exclusion criteria, which are detailed in the clinical research protocol.

[3] Study design

This is a nonrandomized, open-label, stepwise dose escalation study of G47delta. The target population of the study consists of patients with recurrent or progressive glioblastoma, to whom G47delta will be intratumorally administered in a stereotactic manner. The second dose of the same amount of G47delta will be administered into the same site within 5 days to 14 days (7 days to 14 days for the first subject in each cohort). The dose will be increased in three steps per 3 subjects, and once the safety is confirmed, the maximum dose will be administered to 12 additional subjects. The primary objective is to evaluate the safety, or the types and incidence of AEs. The secondary objective is to evaluate the efficacy of G47delta based on the radiographic objective tumor response, overall survival, and progression-free survival.

(2) Subject inclusion/exclusion criteria

1. Inclusion criteria

Patients with histologically confirmed glioblastoma that is unresponsive to radiation therapy, ie, glioblastoma that has recurred or progressed after radiation therapy or is increasing in tumor size during radiation therapy

Patients whose tumor is located at sites that do not meet the exclusion criteria

Patients with enhancing lesion of ≥1.0 cm on MRI within 14 days before G47delta administration

Patients having or not having history of chemotherapy

Patients with Karnofsky Performance Status (KPS) ≥60%, or 50% in the presence of hemiplegia due to surgical tumor resection

Patients aged ≥18 years

Patients with stable steroid regimen (use of steroids is acceptable) for at least 1 week before G47delta administration

Patients willing to use effective barrier contraceptives until at least 6 months after G47delta administration

Patients with life expectancy ≥3 months

Patients whose principal organs function normally (See the exclusion criteria.)

Patients capable and willing to provide written informed consent

2. Exclusion criteria

Medical history

Patients with current or previous cancers other than curative cervical carcinoma in situ and cutaneous basal cell carcinoma or squamous cell carcinoma

Patients with previous encephalitis, multiple sclerosis, or other central nervous system infection

Patients with current or previous positive test result for human immunodeficiency virus

Patients with current or previous alcohol or other drug abuse

Patients with any contraindication for undergoing contrast enhanced MRI (eg, patients with pacemakers, infusion pumps, or allergy to MRI contrast media)

Patients with other medical or mental abnormalities that may make it difficult to comply with the protocol-specified treatment

Tumor site

Patients with extracranial metastases of tumor

Patients with multiple (at least 2) intracranial glioblastoma lesions

Patients with tumor involvement requiring ventricular, brainstem, or posterior fossa inoculation or access through a ventricle in order to deliver G47delta

Patients with subependymal or subarachnoidal dissemination

Laboratory data

Patients with white blood cells $\leq 2.0 \times 10^3/mm^3$, neutrophils $\leq 1.0 \times 10^3/mm^3$, platelets $\leq 100{,}000/mm^3$, hemoglobin (Hb) ≤9.0 g/dL, or international normalized ratio (INR) or partial prothrombin time (PTT) >1.3-fold the normal value

Patients with serum creatinine ≥1.7 mg/dL

Patients with hepatic transaminase (AST or ALT) >4-fold the normal value

Patients with total bilirubin or direct bilirubin >1.5 mg/dL

Complications

Patients with active herpes infection

Patients requiring anti-HSV therapy (acyclovir or valaciclovir) at study initiation

Patients with active, uncontrolled infection for which surgery is not indicated

Patients with uncontrolled or severe heart failure, diabetes mellitus, hypertension, interstitial pneumonia, renal failure, autoimmune disease, etc.

History of allergy

Patients with allergy to anti-HSV drugs (acyclovir)

Concomitant mediation/therapy

Patients who have received other investigational products within 30 days before G47delta administration

Patients who have received immunotherapy (interferon, etc.) within 6 weeks before G47delta administration

Patients who have received a vaccination within 30 days before G47delta administration

Patients who have undergone brain tumor resection within 30 days before G47delta administration

Patients having history of gene therapy or oncolytic virus therapy other than G47delta

Patients having history of or those have already been registered for oncolytic virus therapy with G47delta

Pregnancy-related criteria

Pregnant or nursing women

Others

Patients with other conditions considered inappropriate by the investigator in charge

(3) Method for obtaining consent from subjects

1. Preparation and revision of the informed consent form

   1) In this research, the informed consent form will be prepared in accordance with the form specified by the institution.

   2) The informed consent form should be approved by the institutional review board (IRB) for the clinical research of gene therapy.

   3) Revision of the informed consent form with significant changes requires review and approval by the IRB for the clinical research of gene therapy.

2. Explanation to patients

   Prior to registration, the study doctor will provide the written information for subjects approved by the IRB for the clinical research of gene therapy to each patient and explain the following details verbally in the presence of the clinical research coordinator (CRC).

   1) Name and symptoms of the disease

   2) That this is clinical research

   3) Differences between clinical research and general medical practice

   4) Design and significance of this research

   5) Contents of protocol-specified treatment

      Treatment method, entire protocol-specified treatment period, etc.

   6) Effects expected from the protocol-specified treatment

Survival benefits, objective tumor response, etc.

7) Expected AEs, complications, and sequelae, and how to manage them

   Severity and frequency of expected AEs including complications, sequelae, and related death, and how to manage these events when they occur

8) Cost burden and compensation

   That, if a healthy injury occurs, the study doctor will provide appropriate treatment, but there will be no financial compensation for the injury

9) Alternative treatments

   Contents, effects, adverse reactions, etc. of current general treatments (including palliative care) and standard of care, and advantages and disadvantages of alternative treatments if selected

10) Expected benefits and possible disadvantages for patients from participation in the study

   Potential benefits and possible disadvantages of participating in the study

11) Direct access to medical history

   That relevant parties including members of the Independent Data Monitoring Committee (IDMC) may directly access medical history and other information as necessary with permission from the head of the medical institution

12) Refusal and withdrawal of consent

   That patients can freely refuse to provide consent before participating in the study and withdraw consent even after they provide it without unreasonable loss of medical benefits

13) Protection of human rights

   That maximum efforts will be made to protect confidentiality of the name and other personal information of patients

14) Right to ask questions

   That patients will be informed of contact information of the study doctor and the research director in writing and be entitled to freely ask questions about the study and treatment

3. Consent

1) Method for obtaining consent

   On or after the following day of the explanation of the study, after confirming that the subject has fully understood the contents of the study, the subject will be asked to participate in the study. If the subject has consented to participate in the study, the name of the physician who provided the explanation and the subject who received the explanation and provided consent as well as the date of consent will be provided on the attached consent form with signatures of the physician and the subject.

2) Rules on signature of the substitute writer

If it is difficult for the subject to sign by himself/herself because of neurological symptoms (eg, paralysis, tremor), the name of the subject may be written by a substitute writer (however, the consent itself must be based on the will of the subject). The substitute writer will be designated by the subject from among the following persons: the subject's spouse, adult children, parents, adult siblings or grandchildren, grandparents, relatives living together, or persons considered equivalent to these close relatives.

3) Number of copies of the consent form

Three copies of the consent form will be prepared, including one to be handed to the subject, one to be retained at the Medical Safety Management Department, and one to be retained with the medical chart.

4) Revision of the informed consent form and re-informed consent

When there are efficacy or safety information or changes to the protocol that may affect subjects' consent to participate in the study, the information will be provided to subjects immediately, and the willingness of subjects as to whether to continue their participation in the study will be reconfirmed. At the same time, the informed consent form and other relevant documents will be revised with approval of the IRB for the clinical research of gene therapy to obtain re-informed consent of subjects. After the clinical study is initiated with consent of a subject, if the subject is no longer considered capable of giving consent or approval because of worsening of the pathological condition, etc., his/her legally acceptable representative may decide whether to provide re-informed consent on behalf of the subject.

5) Withdrawal of consent

Subjects may withdraw their consent at any time for any reason. If the subject is no longer considered capable of withdrawing consent because of worsening of the pathological condition, etc., his/her legally acceptable representative may decide whether to withdraw consent on behalf of the subject.

4. Procedures for registration

1) After providing explanations to candidate patients and obtaining their consent, the study doctor will perform the specified tests to collect information necessary for eligibility assessment.

2) After entering the information concerning individual inclusion/exclusion criteria in the subject registration form, the study doctor will present the case to the Eligibility Assessment Committee of the institution to confirm that the patient meets all of the inclusion criteria and none of the exclusion criteria. The assessment by the Eligibility Assessment Committee will then be confirmed by the IDMC.

3) The completed subject registration form will be sent by fax to the data center.

4) The data center will review the information received and then assign a registration number, and specify the dose of G47delta according to the progress of the study.

Subsequently, the registration confirmation form will be prepared and sent to the study doctor. If any inadequacies are found in the received registration form, the data center will contact the study doctor to correct the inadequacies.

(4) Study period and target sample size

1. Study period

The target registration period is approximately 1 year. The observation period is 90 days after G47delta administration. For 2 years after G47delta administration, follow-up will be performed for overall survival and progression-free survival.

Details of observation items are provided in "(5) Method for the clinical research of gene therapy, 4. Laboratory parameters and observation items." Given that no viral shedding was observed at any time point after administration in a nonclinical safety study on intracerebral administration of G207 in monkeys[29] or a Phase 1 clinical study of G207,[30] viral shedding will be tested for 7 days after the second dose.

2. Target sample size

The target sample size is 21 subjects (up to 30 subjects). If no Grade 3 or higher G47delta-related AEs are reported, G47delta will be administered to 9 subjects at the dose escalation phase and to another 12 subjects at the maximum dose, for a total of 21 subjects. The maximum sample size in case Grade 3 or higher G47delta-related AEs occur and additional subjects are enrolled is 30 subjects.

(5) Method for the clinical research of gene therapy

1. Selection of control groups

Since this clinical research will be conducted in an open-label manner, no blinding procedures or control group will be used.

2. Gene transduction method (excluding safety and efficacy issues)

Following the informed consent process, subjects will undergo tests for eligibility assessment and be registered as clinical research subjects.

Rules on prior therapy (Part of inclusion/exclusion criteria are listed again.)

Patients with glioblastoma histologically confirmed by surgery (stereotactic biopsy or surgical resection by craniotomy) at the time of initial onset or recurrence, at least 30 days ago.

Patients who have received radiation therapy, irrespective of radiation method and dose, completion/incompletion of the therapy, and time from completion of the therapy.

There is no restriction with respect to a history of chemotherapy and time from completion of the therapy.

The first dose of G47delta should be administered within 30 days after subject registration. If it is administered after 31 days or later, the reason should be provided in the case report form (CRF).

G47delta will be administered in the operating room in an inpatient setting. For administration, the Leksell stereotactic surgery system will be used to prepare a burr hole under local or general anesthesia and administer G47delta stereotactically into enhancing tumor lesions under MRI guidance. G47delta will be dilated with 10% glycerol/PBS to make a total volume of 1 mL and injected slowly into 2 to 5 parts of the target tumor after biopsy. The second dose of G47delta will be administered in the same way from the same burr hole within 5 days to 14 days after the first dose (7 days to 14 days for the first subject in each cohort). In consideration of the subject's burden and safety, the second dose will be administered at an interval of at least 7 days in the first subject of each cohort and at an interval of at least 5 days in the second and subsequent subjects, but within 14 days after the first dose so that G47delta can be administered from the same surgical wound and so as to avoid difficulty in administration into the same site due to changes in tumor conditions.

In a nonclinical study using murine subcutaneous tumor models to compare a single dose of G207 1×10^7 pfu with six doses (twice/week) of one-tenth of the dose (1×10^6 pfu), 75% of animals (6 of 8 animals) were cured by the latter regimen, whereas none (0 of 8 animals) were cured by the former, indicating the superiority of repeated doses over a single dose.[31] In a Phase 1b clinical study of G207, G207 was intratumorally administered twice (within 7 days). Based on the above results, two-dose treatment was selected for this research. Furthermore, to distribute G47delta into tumor tissues, and since G207 has been stereotactically administered into five parts of the brain tumor in the Phase 1 clinical study of G207, the number of administration sites for this research was set as 2 to 5.

Specimens obtained by biopsy will be divided on the spot. Some specimens will be sent to the laboratory for pathological diagnosis, while others will be sent to the Department of Surgical Neuro-oncology for tests related to this clinical research.

The hospitalization period is defined as the period until the study doctor considers that the subject can be discharged from the hospital.

Dose escalation

The dose will be increased over 3 cohorts each consisting of 3 subjects. G47delta will be administered twice at a dose of 3.0×10^8 pfu, 1.0×10^9 pfu, or 3.0×10^9 pfu; the total dose is 6.0×10^8 pfu, 2.0×10^9 pfu, or 6.0×10^9 pfu per subject. The first subject of each cohort will receive the second dose after a 6-day observation period following the first dose. In addition, before starting treatment in the next subject of the same cohort, there should be an observation period of at least 6 days after the second dose in the

immediately preceding subject. Before proceeding to the next cohort, there should be an observation period of at least 14 days, including the day of administration, after the second dose to the last subject of the immediately preceding cohort, and approval of the IDMC should be obtained.

If none of the subjects in a cohort experience Grade 3 or higher G47delta-related AEs, the next cohort will be started. If there are no Grade 3 or higher G47delta-related AEs at all in the third cohort, 6.0×10^9 pfu will be the maximum dose.

If one subject experiences Grade 3 or higher G47delta-related AEs at a given dose, 3 subjects will be added to the cohort. If none of the additional 3 subjects experience Grade 3 or higher G47delta-related AEs, the next cohort will be started. After subjects are added to the third cohort, if Grade 3 or higher G47delta-related AEs occur in ≤1 of 6 subjects, 6.0×10^9 pfu will be the maximum dose.

If 2 or more subjects experience Grade 3 or higher G47delta-related AEs at a given dose, the 1-level lower dose at that time will be the provisional maximum tolerated dose.

If the provisional maximum tolerated dose is set, subjects will be added to the cohort for a total of 6 subjects. If no more than one subject experiences Grade 3 or higher G47delta-related AEs at the provisional dose, the dose will be the maximum tolerated dose. If 2 or more subjects experience Grade 3 or higher G47delta-related AEs, the 1-level lower dose at that time will be the provisional maximum tolerated dose, and subjects will be added for a total of 6 subjects at the dose.

If 2 or more subjects experience Grade 3 or higher G47delta-related AEs in the cohort of the lowest dose, this clinical research will be terminated.

Once the maximum dose or the maximum tolerated dose is set, additional 12 subjects will be treated at the dose. Of note, if at least one-third of 12 subjects experience Grade 3 or higher G47delta-related AEs at the dose during treatment, the study will be temporarily discontinued, and whether to discontinue or continue the study will be discussed by the IDMC.

Rationale for dose selection

In a nonclinical safety study using mice of A/J strain that are highly susceptible to HSV-1, the safety of single administration of G47delta was confirmed to be at least equivalent to that of single administration of G207. In a Phase 1 clinical study of G207, there were no Grade 3 or higher AEs with intratumoral administration, starting with a single dose of 1×10^6 pfu and up to a single maximum dose of 3×10^9 pfu. In the study, the maximum dose of 3×10^9 pfu, a total volume of 1 mL, was divided and administered to 5 parts of the tumor. Although the safety of G47delta was considered to be at least equivalent to that of G207 in the nonclinical study, given that G47delta has a higher antitumor effect than G207 and that the method for titer determination is different, the starting dose of G47delta in this clinical research was set as one tenth of the maximum dose in the phase 1 study of G207, with the total volume per dose as 1 mL, and it was decided to administer G47delta to 2 to 5 parts of the tumor.

Criteria for changes to the dose and schedule

There will be no dose change, delay, or reduction for individual subjects depending on AEs (see the next section "Discontinuation of protocol-specified treatment").

Discontinuation of protocol-specified treatment

Protocol-specified treatment will be discontinued in any of the following cases. If the treatment is discontinued after it is initiated, recording of the observation items will be continued. The date of discontinuation/completion of protocol-specified treatment will be the date of death if it occurs during the protocol-specified treatment and the date of the decision to discontinue the protocol-specified treatment in other cases. The criterion for the discontinuation of protocol-specified treatment will be entered in the CRF.

[1] In case progression of the primary disease is observed after the start of treatment

Progression of the primary disease includes both radiographic PD and apparent clinical progression. No new treatment is specified for progression of the primary disease.

[2] In case protocol-specified treatment cannot be continued due to AEs

i) Grade 4 nonhematologic toxicities due to G47delta occur (nonhematologic toxicities: AEs other than those in the "Blood/bone marrow" category of the NCI-CTC).

ii) G47delta treatment is discontinued due to AEs during surgery

iii) The study doctor considers it necessary to discontinue treatment due to AEs.

[3] In case the subject requests to withdraw from the protocol-specified treatment or withdraw consent for reasons possibly related to AEs (This category applies if the involvement of AEs cannot be ruled out.)

[4] In case the subject requests to withdraw from the protocol-specified treatment for reasons unrelated to AEs (This category applies only when the involvement of AEs can be ruled out almost certainly, such as relocation of the subject or his/her family.)

[5] Death during protocol-specified treatment

[6] Other cases where the subject is found to be ineligible for the study for reasons such as disease progression during the period after registration until the start of treatment (protocol-specified treatment cannot be initiated because of rapid progression), protocol deviation, or change of the pathological diagnosis after registration, test results, etc. no longer meet the inclusion criteria for reasons such as worsening of complications, or prohibited concomitant therapy is required

[7] Other cases where the study doctor considers it appropriate to discontinue the treatment

3. Pretreatment and concomitant therapy

1) Pretreatment

No pretreatment will be performed.

2) Concomitant therapy

[1] Concomitant use of steroids is allowed. However, the dose from 7 days before eligibility assessment to 7 days after the second dose of G47delta should be stable. If a dose change is clinically required, the reason for the change will be entered in the CRF.

[2] There are no limitations on the use of osmotic diuretics or anticonvulsants.

[3] Antibiotics will be administered in principle during and after surgery, with no limitations on the regimen.

[4] Concomitant use of anti-HSV agents such as acyclovir or valaciclovir (excluding use in subjects with HSV-1 infection after G47delta administration, including suspected cases), non-steroidal immunosuppressants, and immunotherapeutic agents including interferon is prohibited.

[5] If any concomitant medications are used, including commercial medicines, vaccines, and prohibited concomitant drugs, the drug name, dose, dosing frequency, route of administration, date of administration, and reason for use will be entered in the CRF.

3) Supportive therapy

[1] HSV-1 infection-associated encephalitis

If there is persistent fever, seizure, muscle weakness, aphasia, impaired consciousness, or worsening of other neurological symptoms that cannot be accounted for by the primary disease, or inflammation with bleeding or increased peritumoral edema on images, HSV-1 infection-associated encephalitis should be suspected, and the polymerase chain reaction (PCR) test of cerebrospinal fluid (if no increased intracranial pressure is present) or blood, virus cultivation, and if necessary, brain biopsy should be performed. For HSV-1 infection-associated encephalitis, treatment with anti-HSV agents such as acyclovir should be initiated immediately based on the standard therapy for herpes encephalitis.

[2] Other AEs

Other AEs will be treated with appropriate supportive therapy based on the current medical standards.

4) New treatment

[1] After the second G47delta dose, subjects will be observed without other antitumor therapy until progression or recurrence occurs or for 90 days, whichever comes earlier.

[2] No treatment after discontinuation of the protocol-specified treatment or the 90-day observation period is specified.

4. Laboratory parameters and observation items

1) Eligibility assessment after informed consent

[1] Current illness, medical/surgical history

[2] Physical findings, height/body weight

[3] Neurological findings

[4] Vital signs

[5] KPS

[6] Medication history

[7] Blood counts (including differential leukocyte and platelet counts)

[8] Blood chemistry test

Hepatic function (total bilirubin, Al-P, LDH, γGTP, AST, ALT)

Renal function (creatinine)

Electrolytes (Na, K)

[9] Coagulation (PT INR and PTT)

[10] ECG

[11] Chest x-ray

[12] Contrast head MRI

2) From registration up to the day before the first G47delta dose

[1] Lymphocyte CD4 and CD8 counts and CD4/CD8 ratio

[2] Serology including HSV antibody titer (ELISA)

[3] Delayed hypersensitivity skin test

3) Day before the first G47delta dose

[1] Neurological findings

[2] Vital signs

[3] KPS

[4] Blood counts (including differential leukocyte and platelet counts)

[5] Blood chemistry test

Hepatic function (total bilirubin, Al-P, LDH, γGTP, AST, ALT)

Renal function (creatinine)

Electrolytes (Na, K)

[6] Coagulation (PT INR and PTT)

[7] Concomitant medication

[8] Assessment of AEs

4) Before administration on the day of the first G47delta dose

[1] Contrast head MRI

5) During administration on the day of the first G47delta dose

[1] Tumor tissue sampling

6) After administration on the day of the first G47delta dose

[1] Non-contrast head CT

[2] Neurological findings

[3] Vital signs

[4] KPS

[5] Concomitant medication

[6] Assessment of AEs

7) Next day of the first G47delta dose

[1] Non-contrast head CT

[2] Neurological findings

[3] Vital signs

[4] KPS

[5] Concomitant medication

[6] Blood chemistry test

Hepatic function (total bilirubin, Al-P, LDH, γGTP, AST, ALT)

Renal function (creatinine)

Electrolytes (Na, K)

[7] HSV shedding (PCR of saliva and urine; if positive, quantitative PCR will also be performed.)

[8] Serum PCR and virus culture

[9] Assessment of AEs

8) Day before the second G47delta dose

[1] Neurological findings

[2] Vital signs

[3] KPS

[4] Blood counts (including differential leukocyte and platelet counts)

[5] Blood chemistry test

Hepatic function (total bilirubin, Al-P, LDH, γGTP, AST, ALT)

Renal function (creatinine)

Electrolytes (Na, K)

[6] Coagulation (PT INR and PTT)

[7] Concomitant medication

[8] Assessment of AEs

9) Before administration on the day of the second G47delta dose

[1] Contrast head MRI

10) During administration on the day of the second G47delta dose

[1] Tumor tissue sampling

11) After administration on the day of the second G47delta dose

[1] Non-contrast head CT

[2] Neurological findings

[3] Vital signs

[4] KPS

[5] Concomitant medication

[6] Assessment of AEs

12) Next day of the second G47delta dose

[1] Non-contrast head CT

[2] Neurological findings

[3] Vital signs

[4] KPS

[5] Concomitant medication

[6] Blood counts (including differential leukocyte and platelet counts)

[7] Blood chemistry test

Hepatic function (total bilirubin, Al-P, LDH, γGTP, AST, ALT)

Renal function (creatinine)

Electrolytes (Na, K)

[8] HSV shedding (PCR of saliva and urine; if positive, quantitative PCR will also be performed.)

[9] Serum PCR and virus culture

[10] Assessment of AEs

13) 7 days after the second G47delta dose ± 2 days

[1] Neurological findings

[2] Vital signs

[3] KPS

[4] Blood counts (including differential leukocyte and platelet counts)

[5] Blood chemistry test

Hepatic function (total bilirubin, Al-P, LDH, γGTP, AST, ALT)

Renal function (creatinine)

Electrolytes (Na, K)

[6] Coagulation (PT INR and PTT)

[7] HSV shedding (PCR of saliva and urine; if positive, quantitative PCR will also be performed.)

[8] Serum PCR and virus culture

[9] Contrast head MRI

[10] Concomitant medication

[11] Assessment of AEs

14) 28 days after the second G47delta dose ± 4 days

[1] Physical findings, body weight

[2] Neurological findings

[3] Vital signs

[4] KPS

[5] Blood counts (including differential leukocyte and platelet counts)

[6] Blood chemistry test

Hepatic function (total bilirubin, Al-P, LDH, γGTP, AST, ALT)

Renal function (creatinine)

Electrolytes (Na, K)

[7] Coagulation (PT INR and PTT)

[8] Lymphocyte CD4 and CD8 counts and CD4/CD8 ratio

[9] Serology including HSV antibody titer (ELISA)

[10] Delayed hypersensitivity skin test

[11] Contrast head MRI

[12] Concomitant medication

[13] Assessment of AEs

15) 2 months after the second G47delta dose ± 7 days

[1] Physical findings, body weight

[2] Neurological findings

[3] Vital signs

[4] KPS

[5] Blood counts (including differential leukocyte and platelet counts)

[6] Contrast head MRI

[7] Concomitant medication

[8] Assessment of AEs

16) 3 months after the second G47delta dose ± 7 days

[1] Physical findings, body weight

[2] Neurological findings

[3] Vital signs

[4] KPS

[5] Blood counts (including differential leukocyte and platelet counts)

[6] Lymphocyte CD4 and CD8 counts and CD4/CD8 ratio

[7] Delayed hypersensitivity skin test

[8] Serology including HSV antibody titer (ELISA)

[9] Contrast head MRI

[10] Concomitant medication

[11] Assessment of AEs

Study Schedule	Pre-treatment Eligibility Assessment	Pre-treatment Up to the Day before Admin-istration	Week 1 Day before First Dose	Week 1 Day of First Dose	Week 1 Next Day of First Dose	Week 2 Day before Second Dose	Week 2 Day of Second Dose	Week 2 Next Day of Second Dose	Week 3 7 Days after Second Dose	Week 5 1 Month after Second Dose	Week 9 2 Months after Second Dose	Week 13 3 Months after Second Dose
Physical findings												
Informed consent	○											
Medical history, physical findings	○									○	○	○
Vital signs, neurological findings	○		○	○	○	○	○	○	○	○	○	○
KPS	○		○	○	○	○	○	○	○	○	○	○
Assessment of AEs			○	○	○	○	○	○	○	○	○	○
Recording of concomitant medication	○		○	○	○	○	○	○	○	○	○	○
Laboratory findings												
Blood counts, differential leukocyte count	○		○		○	○		○	○	○	○	○
Biochemistry and coagulation	○		○		○	○		○	○	○		
ECG	○											
Non-contrast chest radiography	○											
Lymphocyte subsets		○								○		○
Delayed type skin reaction		○								○		○
HSV antibody titer		○								○		○
HSV shedding (urine, saliva)					○			○	○			
Serum HSV					○			○	○			
Imaging												
Head CT				○	○		○	○				
Head MRI (Gd contrast)	○			○			○		○	○	○	○
Treatment, surgery												
G47delta administration				○			○					
Tumor tissue sampling				○			○					

MRI will be performed before surgery, and CT after surgery, on the day of administration.

5. Expected AEs and how to manage them

1) Procedures for reporting and handling of AEs

The definition of AEs and procedures for reporting and handling of AEs are separately described in the “Procedure for Assessment and Reporting of Adverse Evens.” The description in this section is an excerpt of the procedures.

2) Definition of AEs

An AE is any untoward or intended symptom, sign (including laboratory abnormalities), or disease that occurs during the clinical research, whether or not considered related to the treatment given. AEs do not include symptoms and signs that are related to the primary disease or chronically present prior to G47Δ administration.

3) Definition of serious adverse events (SAEs)

SAEs are defined as the following AEs:

[1] Fatal.

[2] Life-threatening.

[3] Requires inpatient hospitalization or prolongation of existing hospitalization.

[4] Disability.

[5] May result in disability.

[6] Congenital anomaly or birth defect.

[7] Other events that are serious according to the above.

The term “disability” here refers to an event that results in persistent or significant disability/incapacity.

4) Assessment and reporting of AEs

[1] Information of AEs to be recorded in the CRF

AEs that occur will be recorded in the CRF based on the National Cancer Institute Common Terminology Criteria for Adverse Events (NCI-CTCAE) Version 3.0 Japanese version (CTC; available for download at http://www. ccijapan.com, http://www.kutrc.org, or http://www.tri-kobe.org). AEs not listed in the current CTC will also be graded and recorded based on the CTC. Items not provided in the said form will be classified into “Other” disabilities of the relevant category, graded in accordance with the following criteria, and recorded in the CRF.

1. Grade 0: Normal, normal/within reference range (WNL)
2. Grade 1: Mild/mild disorder
3. Grade 2: Moderate/moderate disorder
4. Grade 3: Severe/severe disorder
5. Grade 4: Life-threatening or disabling disorder
6. Grade 5: Death

All AEs will be followed up with necessary tests and recording of the test results until the condition of the subject returns to the pre-event condition or until the study doctor considers that the event has resolved satisfactorily.

[2] Assessment of causality

The study doctor will assess the relationship between G47delta and AEs and record the assessment based on the following criteria:

1. Definitely related: There is a clinically reasonable temporal relationship between G47delta administration and the onset of the AE, and other possibilities are ruled out.
2. Probably related: There is a clinically reasonable temporal relationship between G47delta administration and the AE. The AE is unlikely to be due to the primary disease, complications, other drugs, or other procedures (only if applicable; the time course is such that the AE also improves after G47delta is discontinued).
3. Possibly related: There is or is not a clinically reasonable temporal relationship between G47delta administration and the onset of the AE. A causative role of the study drug cannot be excluded.
4. Probably unrelated: Another factor than G47delta is a likely to be the cause of the AE. There is no temporal relationship between G47delta administration and the onset of the AE. The causality is unlikely from medical and biological perspectives.
5. Unrelated: There is no temporal relationship between G47delta administration and the onset of the AE. The AE is likely to be due to the primary disease, complications, other drugs, or other procedures. The AE does not improve even after discontinuation of G47delta.

5) Test abnormalities

1. Clinically insignificant test abnormalities

All test results will be recorded in the CRF and managed. Clinically insignificant test abnormalities, ie, slight changes from the normal value not requiring medical procedures, are not regarded as AEs.

2. Clinically significant test abnormalities

Abnormalities of CTC Grade 3 or 4 and those considered clinically significant by the study doctor will be recorded in the CRF. In addition, abnormal values not related to already reported AEs, diseases, or complications, and those requiring change of drugs to be concomitantly used will be recorded as AEs. For these abnormal values, retest should be performed to immediately assess the "seriousness." Value that meet the definition of "serious" should be reported as instructed in the section of SAEs.

6) Expected AEs

Expected AEs of intratumoral administration of G47delta include the following:

[1] AEs due to administration of the study drug G47delta

1. Symptoms of systemic viral infection such as rigors/chills, myalgia, arthralgia, or lymphadenopathy
2. Allergic reactions such as itching, urticaria, blood pressure fluctuation, or dyspnea
3. Symptoms of HSV-1 encephalitis such as fever, seizure, muscular weakness, aphasia, or consciousness disturbance
4. Headache

[2] AEs related to the primary disease

1. Development or worsening of consciousness disturbance or neurological symptoms
2. Seizure
3. Intracranial hypertension symptoms such as headache, queasy, or vomiting

[3] AEs related to surgical procedure

1. Development or worsening of consciousness disturbance or neurological symptoms
2. Intracerebral hemorrhage or intratumoral hemorrhage
3. Postoperative infections such as meningitis or wound infection
4. Seizure
5. Systemic postoperative complications such as pneumonia or liver dysfunction
6. CSF leak

7) Expedited reporting of AEs and measures to be taken

[1] AEs with reporting obligations

AEs with reporting obligations are "SAEs" defined in the "Definition" that occur during the clinical research or the observation period.

[2] Reporting procedures

1. Primary reporting (promptly within 72 hours of learning of the occurrence)

In the event of an AE with reporting obligations, the research director will perform first reporting to the head of his/her medical institution, responsible person of the IRB for the clinical research of gene therapy of the medical institution, responsible persons of the IRBs for the clinical research of gene therapy of other medical institutions participating in this clinical research, and the IDMC within 72 hours of learning of the occurrence of the AE, irrespective of the causal relationship with study treatment. Reporting should be performed verbally, by telephone, or by e-mail and the report should be submitted in person, by fax, or by e-mail with the information known up to that time point on the "Report on Serious Adverse Events."

The head of the medical institution will promptly inform the Minister of the Ministry of Health, Labour and Welfare (MHLW) and the Minister of the

Ministry of Education, Culture, Sports, Science and Technology (MEXT) of a summary of the AE and measures to be taken.

2. Secondary reporting (within 15 days of learning of the occurrence)

The research director will complete the "Report on Serious Adverse Events" and submit it to the head of his/her medical institution, responsible person of the IRB for the clinical research of gene therapy of the medical institution, and responsible persons of the IRBs for the clinical research of gene therapy of other medical institutions participating in this clinical research in person, by fax, or by mail within 7 days of learning of the occurrence of the SAE.

The head of the medical institution will report to the Minister of the MHLW and the Minister of the MEXT with the attached Form 5 of the "Guideline for Gene Therapy Clinical Studies" within approximately 15 days of learning of the occurrence.

3. Detailed reporting of investigation

If detailed information is requested by the IDMC, the research director and the data center will conduct necessary and sufficient investigation according to the instructions and submit the report.

4. Final reporting

As soon as the outcome of the SAE is confirmed, the research director will prepare a report on the course and outcome after secondary reporting and submit it to the head of his/her medical institution. The head of the medical institution will report it to the Minister of the MHLW and the Minister of the MEXT.

5. Measures to be taken after final reporting

The research director will send the final report to the IDMC members and the data center. In addition, if the research director considers it necessary, he/she will request re-assessment to the IDMC. Ongoing AEs other than those "definitely unrelated to G47delta" as of the last follow-up day will continue to be followed up.

6. Evaluation method, evaluation criteria, and discontinuation criteria for the clinical research of gene therapy

1) Evaluation method and criteria

(a) Endpoints

Primary endpoint: Safety evaluation (type, frequency, and severity of adverse events [AEs]).

Secondary endpoints: Objective tumor response during the 90-day observation period after G47delta administration. Overall survival and progression-free survival.

(b) Response assessment

The objective tumor response will be assessed in accordance with the assessment criteria of the WHO based on MRI images, as follows:

CR: Complete Response

Complete disappearance of a target lesion

PR: Partial Response

A decrease in the sum of areas of target lesions by at least 50%, with no appearance of new lesions

SD: Stable Disease

A decrease in the sum of areas of target lesions by less than 50% or an increase in the sum of areas of target lesions by less than 25%, with no appearance of new lesions

PD: Progressive Disease

An increase in the sum of areas of target lesions by at least 25%, or appearance of new lesion(s)

NE: Not Evaluable

The incidence of AEs will be calculated with the number of all treated subjects as the denominator. In addition, for all treated subjects and subjects treated at the maximum dose, the radiographic response proportion at 90 days after G47delta administration will be calculated. Overall survival and progression-free survival will be calculated for the follow-up period.

(c) Incidence of AEs

For each of the AEs listed below that occur up to 90 days after the second dose of G47delta, the frequency of the worst grade event based on the NCI-CTCAE Version 3.0 (Japanese translation, JCOG/JSCO version) will be calculated (by group) using the number of patients who received at least part of the protocol-specified treatment (all treated patients), regardless of whether they are eligible or ineligible, as the denominator.

[1] Blood/bone marrow: Hemoglobin, leukocytes (total WBC), platelets

[2] Metabolism/laboratory data: Total bilirubin, alkaline phosphatase, LDH, GGT (γ-glutamyl transpeptidase), AST/SGOT (serum glutamic oxaloacetic transaminase), ALT/SGPT (serum glutamic pyruvic transaminase), Na, K, creatinine

[3] Constitutional symptoms: Fever (in the absence of neutropenia, where neutropenia is defined as $ANC<1.0 \times 10^9$ /L), malaise, myalgia, headache, anorexia, nausea, vomiting

[4] Neurology: Consciousness disturbance, neurological symptoms, seizure

[5] Infection: Infection in the absence of neutropenia

[6] Central nervous system complications: Encephalitis, meningitis, intracerebral hemorrhage, intratumoral hemorrhage, hydrocephalus

(d) Incidence of SAEs

The incidence of SAEs will be calculated using the number of patients who started at least part of the protocol-specified treatment (all treated patients) as the denominator and the number of patients who experienced any of the SAEs listed below at least once as the numerator.

[1] All deaths (regardless of the cause of death or the causal relationship with treatment) during the period from the first dose of G47delta to within 30 days after the second dose of G47delta

[2] Deaths that occur 31 days or later after the second dose of G47delta for which the causal relationship with treatment cannot be ruled out

[3] Grade 4 AEs

(e) Overall survival

Time to death from the day of the first surgery (regardless of the cause of death). Surviving subjects will be censored on the last date when their survival was confirmed. Subjects lost to follow-up will be censored on the last date when their survival was confirmed before being lost to follow-up.

(f) Progression-free survival (PFS)

Time to the assessment of disease progression or death due to any cause, whichever comes earlier, from the day of the second dose of G47delta. "Disease progression" includes both radiographic PD and worsening of the primary disease that cannot be confirmed by imaging (clinical progression). Surviving subjects without disease progression will be censored on the last date when the absence of clinical progression was confirmed (date of the final confirmation of progression-free survival).

(g) Response proportion (response rate)

Percentage of subjects with the "response" of either CR or PR among eligible subjects with measurable lesions.

7. Ensuring of subject safety and compensation for health injuries

1) Monitoring

1. IDMC

The IDMC will be established under the IRB for the clinical research of gene therapy. The chairperson of the IRB for the clinical research of gene therapy will select at least three members from among not belonging to the main research body. The members will include a physician familiar with the treatment of malignant brain tumor, statistics expert, and person with expertise in AE assessment. The IDMC has the following roles:

[1] Review of the assessment by the Eligibility Assessment Committee

[2] Confirmation of the safety and efficacy assessment and decision on the appropriateness of dose escalation (between cohorts)

[3] Confirmation of the safety and efficacy assessment and decision on the appropriateness of the maximum dose or the maximum tolerated dose (at the end of dose escalation)

[4] Receipt of reports on "SAEs" and assessment of the causal relationship with this clinical research

The IDMC will examine the need for protocol revision with respect to the following items, and if necessary, may exert the authority to recommend the research director to revise the protocol or discontinue the study.

[1] Change of the registration period

[2] Changes to the eligibility criteria

[3] Resetting of the target sample size

[4] Changes to the protocol-specified treatment plan

[5] Other necessary changes

2. Eligibility Assessment Committee

The Eligibility Assessment Committee will be established under the IRB for the clinical research of gene therapy. The Eligibility Assessment Committee will review and verify that target patients meet all of the inclusion criteria and none of the exclusion criteria. Meetings of the Eligibility Assessment Committee will be held as a regular conference at 9:00 am every Monday at the Department of Surgical Neuro-oncology of the IMSUT Hospital. In the meetings, the research director or the study doctor will provide case presentation, but will not be involved in the eligibility assessment. The record of approval by the Eligibility Assessment Committee will be documented in the Case Registration Form.

2) Review by the IRB for the clinical research of gene therapy

Prior to subject registration, the protocol will be reviewed and approved by the IRB for the clinical research of gene therapy of the study site.

3) Rules to be complied with

[1] All investigators involved in this research will conduct it in accordance with the Declaration of Helsinki of the World Medical Association (http://www.med.or.jp/wma/).

[2] This research will be conducted based on the Ministerial Ordinance on Good Clinical Practice (GCP) for Drugs (Ministry of Health and Welfare Ordinance No. 28, 1997): http://law.e-gov.go.jp/htmldata/H09/H09F03601000028.html).

[3] For this research, an application for Type 1 Use regulations based on the "Law for Securing Multiplicity of Living Organisms under the Use Control of Genetically-Engineered Living Organisms" (http://www.mhlw.go.jp/general/seido/kousei/i-kenkyu/seibutu/tuuchi.html) will be submitted, and the law will be complied with.

[4] This research will be conducted in accordance with the Guideline for Gene Therapy Clinical Studies (27 Mar 2002 [fully revised on 28 Dec 2004] [partially revised on 01 Dec 2008]: http://www.mhlw.go.jp/general/seido/kousei/i-kenkyu/idenshi/0504sisin.html).

4) Compliance with the protocol

Investigators participating in this research will comply with the protocol of this research as long as the safety and human rights of subjects are not compromised. However, clinical measures to protect the health and life of patients will be prioritized.

5) Cost burden for the clinical research

[1] Funding source and financial relationship

This research will be conducted with support of the MEXT's "Coordination, Support and Training Program for Translational Research." The research director has the right to obtain a patent for the study drug G47delta in Japan.

[2] Costs for the clinical research

The study drug G47delta will be supplied free of charge by the IMSUT Hospital. Costs of the tests performed at the Department of Surgical Neuro-oncology of the IMSUT Hospital will be covered by the research funds, etc. No payments will be made for transportation fees or other rewards.

6) Compensation for health injuries

[1] In the event of a healthy injury due to this research, treatment fees during the acute-phase and the period until symptoms become stable or cured, testing costs, and hospitalization costs will be borne by the research group. However, the period covered is up to 1 year after treatment.

[2] Actual costs other than medical fees and costs including treatment fees after symptoms due to adverse drug reactions, etc. become stable will not be compensated.

[3] The above [1] and [2] apply to treatments provided at other medical institutions.

[4] The investigator and the study doctor will purchase liability insurance in preparation for compensation.

7) Revision of the protocol

[1] Reporting of protocol revision

If the protocol is revised, all changes and reasons for the changes will be reported to the IRB for the clinical research of gene therapy of the study site, irrespective of the significance of the changes.

Based on the report to the IRB for the clinical research of gene therapy, the head of the medical institution will report the changes to the protocol to the Minister of the MHLW and the Minister of the MEXT with the attached Form 2 of the "Guideline for Gene Therapy Clinical Studies."

[2] Protocol revision requiring review

Changes that are considered significant must be reexamined and approved by the IRB for the clinical research of gene therapy. Changes considered significant include those that may increase the risk of study subjects and those to any of the following:

Study design, inclusion criteria, treatment plan, endpoints, target sample size, and expected AEs

[3] Revision of the informed consent form

The informed consent form will also be revised according to the changes to the protocol.

[4] Modification of the CRF

If inadequacies are found after study initiation, including lack of data elements required for the CRF and inappropriate categorization, the CRF will be modified at the research director's discretion as long as the elements to be modified are within the scope of data to be collected specified in "4. Laboratory parameters and observation items" and modification of the CRF is not considered to increase medical and financial burden on registered patients. Modification of the CRF not requiring revision of the text of the protocol will not be handled as protocol revision, but will be reported to the IRB for the clinical research of gene therapy.

8. Completion and early termination of the study

1) Completion of the study

[1] New registration will be terminated when the target sample size is achieved, and this clinical research will be terminated when the observation period has been completed for all enrolled subjects and CRFs have been submitted.

[2] The research director will report the completion of the study to the head of his/her medical institution and the IRB for the clinical research of gene therapy of the medical institution. The head of the medical institution will report the completion of the study to the Minister of the MHLW and the Minister of the MEXT with the attached Form 4 of the "Guideline for Gene Therapy Clinical Studies."

2) Early termination of the study

Early termination refers to discontinuing the clinical research earlier than scheduled based on the any of the following decisions by the IDMC:

[1] Based on information on SAEs or information from other sources than this clinical research, the research is considered to have safety problems.

[2] It is considered difficult to complete the clinical research for reasons such as delayed patient enrollment or frequent protocol deviations.

[3] Early termination based on the number of AEs under observation is described in (5).2.7) [6] and (5).2.7) [7].

[4] The head of the medical institution will report the discontinuation of the study to the Minister of the MHLW and the Minister of the MEXT with the attached Form 3 of the "Guideline for Gene Therapy Clinical Studies."

9. Research organization

1) Research director: Tomoki Todo

Professor, Division of Innovative Cancer Therapy (Department of Surgical Neuro-oncology), Advanced Clinical Research Center, Institute of Medical Science, the University of Tokyo

4-6-1 Shirokanedai, Minato-ku, Tokyo 108-8639, Japan

2) Eligibility Assessment Committee: (See Section 8 (5).7 Ensuring of subject safety and compensation for health injuries)

   [Omitted]

3) Data center:

Established in the Translational Research Center of the University of Tokyo Hospital.

4) CRC (TRC):

Established in the Medical Safety Management Division of the IMSUT Hospital.

5) Author of the protocol:

Tomoki Todo, Professor, Division of Innovative Cancer Therapy (Department of Surgical Neuro-oncology), Advanced Clinical Research Center, Institute of Medical Science, the University of Tokyo

6) Person responsible for statistical analysis:

Hiroshi Ohtsu, Specially Appointed Professor, Department of Clinical Trial Data Management, the Graduate School of Medicine, University of Tokyo

Upon completion of the study, the person responsible for statistical analysis will prepare a statistical analysis plan and a plan for statistical analysis tables/figures and perform statistical analysis based on the plans. In addition, the person responsible for statistical analysis will compile the results in a statistical analysis report.

7) IDMC: (See Section 8 (5).7 Ensuring of subject safety and compensation for health injuries)

Chairperson	Shoji Tsuji	Professor, Department of Neurology, University of Tokyo Hospital
Members	Kazuhiro Nomura	Director Emeritus, Tokyo Rosai Hospital
	Keiichi Nakagawa	Associate Professor, Department of Radiology, University of Tokyo Hospital
	Hiroshi Oyama	Professor, Department of Clinical Information Engineering, the Graduate School of Medicine, University of Tokyo

8) Clinical research facility:

- IMSUT Hospital

  (Newly added for this product in response to transfer of the research director and the clinical research team.)

- University of Tokyo Hospital

  (Already approved for this protocol.)

Research director:

Tomoki Todo

10. Protection of subject privacy and confidentiality

Subject privacy and confidentiality will be protected in compliance with "Rules for Appropriate Control of Personal Information Retained by the IMSUT Hospital." Key elements of the document are summarized below.

(1) Safety control measures at the study site

[1] Administrative system

(i) The personal information protection manager (hereinafter referred to as the "protection manager") will be established in the IMSUT Hospital, which will be undertaken by the hospital director.

(ii) At least one assigned person for personal information protection (hereinafter referred to as "protection staff") will be established in the IMSUT Hospital, which will be undertaken by the person(s) appointed by the protection manager.

(iii) The Personal Information Protection Promotion Committee (hereinafter referred to as the "Committee") will be established as an organization under the direct control of the protection manager.

[2] Responsibilities of the personnel

The personnel (including graduate students, students, and external contracted employees that may handle retained personal information; the same applies hereinafter) must manage and handle retained personal information in an appropriate manner by following the provisions of relevant laws and rules, etc. and instructions of the general protection manager, protection manager, and protection staff in line with the spirit of the laws.

[3] Management and handling of retained personal information

(i) The personnel must appropriately manage and handle personal information generated or obtained while on duty with attention to the risk of personal information leakage, etc.

(ii) The personnel must not handle retained personal information for other purposes than their duties.

(iii) It is prohibited to take retained personal information out of the hospital or keep it after transferring or retiring from the hospital.

(iv) Even when handling retained personal information for the purposes of their duties, the personnel must follow the instructions of the protection manager with regard to reproduction, transmission, and taking out and shipping to outside.

(v) In accordance with the instructions of the protection manager, the personnel will keep media containing retained personal information in a designated place, and when deemed necessary, store the media in a fireproof safe under lock and key.

(vi) When retained personal information or media containing retained personal information are no longer needed, the personnel will erase the information or dispose of the media in a way that makes it impossible to restore or read the information or media, in accordance with the instructions of the protection manager.

(vii) To prevent leakage, loss, and damage of retained personal information by computer viruses, the protection manager will take necessary measures to prevent computer virus infection.

[4] Provision of retained personal information

When providing retained personal information to other persons than administrative organs and incorporated administrative agencies based on Article 9, Paragraph 2, Item 3 and Item 4 of the law, the protection manager will, in principle, exchange a written document with the recipient regarding the purpose of use at the recipient, legal basis for relevant duties, scope and items of records to be used, form of use, etc.

[5] Reporting of incidents and measures to prevent recurrence

In the event of an incident such as leakage of retained personal information that poses a problem in terms of security, the personnel who become aware of the fact will promptly report the incident to the protection manager who manages the relevant retained personal information. The protection manager will take necessary measures to contain or repair the damage.

[6] Publication

Depending on the nature and impact of the incident, the protection manager will implement measures including publication of the fact and measures to prevent recurrence and response to the person involved in the incident, after consulting with the general protection manger.

(2) Protection of personal information in this research

A. Definition of personal information

The term "personal information" in this clinical research refers to information based on the "Act on the Protection of Personal Information" (Act No. 57 of 2003) (hereinafter referred to as "Personal Information Protection Law"), "Guideline for Appropriate Handling of Personal Information by Medical Care and Nursing Care Providers" (dated 24 Dec 2004; MHLW), and "Guideline for Gene Therapy Clinical Studies" (Notification No. 1 dated 27 Mar 2002 by the MEXT and the MHLW), which can identify specific individuals by name, date of birth, medical chart number, and other descriptions included in the relevant treatment information. In this clinical research, which is not intended to reveal genetic information, no genetic information of individuals will be identified.

B. Specification and notification of the purpose of use of personal information

The purpose of use of personal information in this clinical research is based on this protocol prepared in accordance with the "Guideline for Gene Therapy Clinical Studies" and the "purpose of research" provided in the

"Informed Consent Form." For changes to the purpose of use outside the scope permitted under the provisions of Article 15, Paragraph 2 of the Personal Information Protection Law, subjects will be notified and asked to provide consent in writing again.

C. Ensuring of data accuracy

Personal information including data on treatment results will always be reviewed by the "IDMC," and efforts will be made to ensure that the data are accurate and update.

D. Restrictions on provision to third parties

Personal information will not be provided to external parties. If information must be provided for research/analysis purposes for unavoidable reasons, written notice will be issued again to obtain consent.

E. Retention of records

The research director will retain all documents related to the conduct of the study, etc. (application dossiers, notifications, application forms and reports, subject identification number list, consent form, CRF, and documents and records necessary to guarantee the reliability of data, or their copies) for at least 5 years after the end of the study. The person responsible for retention is the research director or the person appointed by the research director.

11. Method for publication of results

(1) The research director may publish the results of this clinical research in scientific journals and at academic meetings. When publishing the results, consideration must be given to the protection of personal information. Research results will belong to the research director. The information obtained in this clinical research may be used for the development of pharmaceutical products of G47delta, and the contents may be disclosed to government agencies of different countries. The above also applies if the study is discontinued or temporarily discontinued in the middle.

(2) Results obtained in the clinical research will not be disclosed to third parties, except in cases where the results are published in accordance with the above procedures.

(3) This research will be registered as clinical research in the Clinical Trials Registry of the University hospital Medical Information Network (UMIN) Center.

REFERENCES

1. Todo T, Martuza, RL, Rabkin, SD, et al. Oncolytic herpes simplex virus vector with enhanced MHC class I presentation and tumor cell killing. Proc Natl Acad Sci USA 98: 6396-6401.2001.
2. CBTRUS (Central Brain Tumor Registry of the United States) Statistical Report: Primary Brain Tumors in the United States 1998-2002. Chicago; Central Brain Tumor Registry of the United States.2005.
3. The Committee of Brain Tumor Registry of Japan: Report of brain tumor registry of Japan (1969-1996) 11th edition. Neurol medico-chirurgica 43 (suppl): 1-25, 2003.
4. Yamaura A (chief editor), Yoshida J (technical editor), eds. Principles and Advances in Neurological Surgery 6, Brain Tumor I, 298-307, Tokyo, Nakayama Shoten Co., Ltd., 2004
5. Walker MD, Strike, TA, Sheline, GE. An analysis of dose-effect relationship in the radiotherapy of malignant gliomas. Int J Radiat Oncol Biol Phys 5: 1725-1731.1979.
6. Stupp R, Mason, WP, van den Bent, MJ, et al. Radiotherapy plus concomitant and adjuvant temozolomide for glioblastoma. N Engl J Med 352: 987-996.2005.
7. Takakura K, Abe, H, Tanaka, R, et al. Effects of ACNU and radiotherapy on malignant glioma. J Neurosurg 64: 53-57.1986.
8. Tanaka M, Ino, Y, Nakagawa, K, et al. High-dose conformal radiotherapy for supratentorial malignant glioma: a historical comparison. Lancet Oncol 6: 953-960.2005.
9. Todo T, Ebright, MI, Fong, Y, et al. Oncolytic herpes simplex virus (G207) therapy for cancer: from basic to clinical. In Tumor Suppressing Viruses, Genes, and Drugs - Innovative Cancer Therapy Approaches. Maruta H. eds., pp45-75, Academic Press, San Diego. 2001
10. Hunter WD, Martuza, RL, Feigenbaum, F, et al. Attenuated, replication-competent herpes simplex virus type 1 mutant G207: safety evaluation of intracerebral injection in nonhuman primates. J. Virol. 73: 6319-6326.1999.
11. Kamei S, Takasu, T. Nationwide survey of the annual prevalence of viral and other neurological infections in Japanese inpatients. Intern Med 39: 894-900.2000.
12. Whitley RJ, Lakeman, F. Herpes simplex virus infections of the central nervous system: therapeutic and diagnostic considerations. Clin Infect Dis 20: 414-420.1995.
13. Wald A, Corey L. Persistence in the population: epidemiology, transmission. In Human Herpesviruses. Arvin A., et al. eds., pp659-671, Cambridge University Press, Cambridge 2007
14. Mori I, Nishiyama, Y, Yokochi, T, et al. Olfactory transmission of neurotropic viruses. J Neurovirol 11: 129-137.2005.
15. Assar, S. et al. Survival of Microorganisms in the Environment. In *Disinfection, Sterilization, and Preservation.* 5th edn Block S. ed., 1221-1242, Lippincott Williams & Wilkins, Philadelphia (2001)

16. Croughan WS, Behbehani, AM. Comparative study of inactivation of herpes simplex virus types 1 and 2 by commonly used antiseptic agents. J Clin Microbiol 26:213-215.1988.

17. Chou J, Kern, ER, Whitley, RJ, et al. Mapping of herpes simplex virus-1 neurovirulence to $\gamma_1$34.5, a gene nonessential for growth in culture. Science 250: 1262-1266.1990.

18. Farassati F, Yang, AD, Lee, PW. Oncogenes in Ras signalling pathway dictate host-cell permissiveness to herpes simplex virus 1. Nat Cell Biol 3: 745-750.2001.

19. Mineta T, Rabkin, SD, Yazaki, T, et al. Attenuated multi-mutated herpes simplex virus-1 for the treatment of malignant gliomas. Nat Med 1: 938-943.1995.

20. Fukuhara H, Martuza, RL, Rabkin, SD, et al. Oncolytic herpes simplex virus vector G47Δ in combination with androgen ablation for the treatment of human prostate adenocarcinoma. Clin Cancer Res 11: 7886-7890.2005.

21. Hoffmann D, Wildner O. Comparison of herpes simplex virus- and conditionally replicative adenovirus-based vectors for glioblastoma treatment. Cancer Gene Ther. 14: 627-639. 2007

22. Liu R, Martuza, RL, Rabkin, SD. Intracarotid delivery of oncolytic HSV vector G47Δ to metastatic breast cancer in the brain. Gene Ther 12: 647-654.2005.

23. Liu R, Varghese, S, Rabkin, SD. Oncolytic herpes simplex virus vector therapy of breast cancer in C3(1)/SV40 T-antigen transgenic mice. Cancer Res 65:1532-1540.2005.

24. Messerli SM, Prabhakar, S, Tang, Y, et al. Treatment of schwannomas with an oncolytic recombinant herpes simplex virus in murine models of neurofibromatosis type 2. Hum Gene Ther 17: 20-30.2006.

25. Todo T, Rabkin, SD, Sundaresan, P, et al. Systemic antitumor immunity in experimental brain tumor therapy using a multimutated, replication-competent herpes simplex virus. Hum Gene Ther 10: 2741-2755.1999.

26. Todo T, Rabkin, SD, Chahlavi, A, et al. Corticosteroid administration does not affect viral oncolytic activity, but inhibits antitumor immunity in replication-competent herpes simplex virus tumor therapy. Hum Gene Ther 10: 2869-2878.1999.

27. Lopez C. Genetics of natural resistance to herpesvirus infections in mice. Nature 258: 152-153.1975.

28. Sundaresan P, Hunter, WD, Martuza, RL, et al. Attenuated, replication-competent herpes simplex virus type 1 mutant G207: safety evaluation in mice. J Virol 74: 3832-3841.2000.

29. Todo T, Feigenbaum, F, Rabkin, SD, et al. Viral shedding and biodistribution of G207, a multimutated, conditionally replicating herpes simplex virus type 1, after intracerebral inoculation in aotus. Mol Ther 2: 588-595.2000.

30. Markert JM, Medlock, MD, Rabkin, SD, et al. Conditionally replicating herpes simplex virus mutant, G207 for the treatment of malignant glioma: results of a phase I trial. Gene Ther 7: 867-874.2000.

31. Chahlavi A, Rabkin, S, Todo, T, et al. Effect of prior exposure to herpes simplex virus 1 on viral vector-mediated tumor therapy in immunocompetent mice. Gene Ther. 6: 1751-1758.1999.
