# Supplementary material for: A phase I/II study of triple-mutated oncolytic herpes virus G47∆ in patients with progressive glioblastoma
Source: Nat Commun. 2022 Jul 21;13:4119. doi: 10.1038/s41467-022-31262-y (PMC9304402; doi:10.1038/s41467-022-31262-y)
Supplement: Supplementary file 3 — Reporting Summary [file 41467_2022_31262_MOESM3_ESM.pdf]

nature research

Corresponding author(s): Tomoki Todo

Last updated by author(s): Jun 8, 2022

Reporting Summary

Nature Research wishes to improve the reproducibility of the work that we publish. This form provides structure for consistency and transparency in reporting. For further information on Nature Research policies, see our Editorial Policies and the Editorial Policy Checklist.

Statistics

For all statistical analyses, confirm that the following items are present in the figure legend, table legend, main text, or Methods section.

n/a	Confirmed	
☐	☒	The exact sample size (n) for each experimental group/condition, given as a discrete number and unit of measurement
☐	☒	A statement on whether measurements were taken from distinct samples or whether the same sample was measured repeatedly
☒	☐	The statistical test(s) used AND whether they are one- or two-sided<br>*Only common tests should be described solely by name; describe more complex techniques in the Methods section.*
☐	☒	A description of all covariates tested
☒	☐	A description of any assumptions or corrections, such as tests of normality and adjustment for multiple comparisons
☒	☐	A full description of the statistical parameters including central tendency (e.g. means) or other basic estimates (e.g. regression coefficient) AND variation (e.g. standard deviation) or associated estimates of uncertainty (e.g. confidence intervals)
☒	☐	For null hypothesis testing, the test statistic (e.g. F, t, r) with confidence intervals, effect sizes, degrees of freedom and P value noted<br>*Give P values as exact values whenever suitable.*
☒	☐	For Bayesian analysis, information on the choice of priors and Markov chain Monte Carlo settings
☒	☐	For hierarchical and complex designs, identification of the appropriate level for tests and full reporting of outcomes
☒	☐	Estimates of effect sizes (e.g. Cohen's d, Pearson's r), indicating how they were calculated

Our web collection on statistics for biologists contains articles on many of the points above.

Software and code

Policy information about availability of computer code

Data collection	No software was used for data collection.
Data analysis	All data analyses were done with IBM SPSS Statistics version 22 software (IBM Corporation, Somers, USA).

For manuscripts utilizing custom algorithms or software that are central to the research but not yet described in published literature, software must be made available to editors and reviewers. We strongly encourage code deposition in a community repository (e.g. GitHub). See the Nature Research guidelines for submitting code & software for further information.

Data

Policy information about availability of data

All manuscripts must include a data availability statement. This statement should provide the following information, where applicable:

- Accession codes, unique identifiers, or web links for publicly available datasets
- A list of figures that have associated raw data
- A description of any restrictions on data availability

All requests for raw and analyzed data will be reviewed and need approval by the Institute of Medical Science Hospital, the University of Tokyo and the University of Tokyo Hospital. Patient-related data not included in the paper were generated as part of a clinical trial and are subject to patient confidentiality. All data shared will be de-identified. Requests should be made to the corresponding author. The study protocol is available as Supplementary Note 1 in the Supplementary Information file. The remaining data are available within the Article, Supplementary Information or Source Data file.

Field-specific reporting

Please select the one below that is the best fit for your research. If you are not sure, read the appropriate sections before making your selection.

- [x] Life sciences
- [ ] Behavioural & social sciences
- [ ] Ecological, evolutionary & environmental sciences

For a reference copy of the document with all sections, see nature.com/documents/nr-reporting-summary-flat.pdf

Life sciences study design

All studies must disclose on these points even when the disclosure is negative.

Sample size	The target sample size planned was 21 subjects (up to 30 subjects) based on the presence of absence of G47Δ-attributable Grade ≥3 adverse events in the initial three cohorts of the dose escalation phase. (According to the decision of IDMC, the actual sample size was 13.) Initially, three cohorts of 3 patients each (9 patients total) were included in determining the safe set dose (phase I part), with an additional 12 patients at the set dose or MTD (phase II part) to reach a target sample size of 21 patients (maximum 30 patients). After the second cohort of the phase I part (6 patients), the IDMC decided to use 1 × 10^9 pfu/dose (total 2 × 10^9 pfu) as the set dose to proceed to the phase II part of this study.
Data exclusions	Patients were excluded based on a priori criteria before the start of the study. There were no data excluded for enrolled patients.
Replication	Findings were from a cohort of patients with glioblastoma and only 1 patient remained alive at final follow-up; replication in this cohort is therefore impossible.
Randomization	This was a phase I/II open-label study without randomization.
Blinding	Blinding not conducted. Open-label study design using surgical patients made blinding not relevant.

Reporting for specific materials, systems and methods

We require information from authors about some types of materials, experimental systems and methods used in many studies. Here, indicate whether each material, system or method listed is relevant to your study. If you are not sure if a list item applies to your research, read the appropriate section before selecting a response.

Materials & experimental systems

n/a	Involved in the study	
[ ]	[x]	Antibodies
[ ]	[x]	Eukaryotic cell lines
[x]	[ ]	Palaeontology and archaeology
[x]	[ ]	Animals and other organisms
[ ]	[x]	Human research participants
[ ]	[x]	Clinical data
[x]	[ ]	Dual use research of concern

Methods

n/a	Involved in the study	
[x]	[ ]	ChIP-seq
[x]	[ ]	Flow cytometry
[ ]	[x]	MRI-based neuroimaging

Antibodies

Antibodies used	Antibodies used<br>Anti-CD4 (rabbit) (clone EPR6855, abcam, Cat. ab133616, Lot. GR3276764-5, dilution 1:250)<br>Anti-CD8 (rabbit) (clone SP16, abcam, Cat. ab101500, Lot. 9116S1711F, dilution 1:100)<br>Anti-HSV-1 (rabbit) (polyclonal, Gene Tex, Cat. GTX73373, Lot. 822100458, ready-to-use)<br>Anti-IDH1 R132H (mouse) (clone H09, dianova, Cat. DIA-H09, Lot. 211129/02, dilution 1:100)<br>Anti-MGMT (mouse) (clone MT3.1, abcam, Cat. ab39253, Lot. GR3422909-2, dilution 1:100)
Validation	All antibodies were purchased directly from manufacturers and the validation statements are available on website of the manufacturers shown below. The application of all antibodies followed the instructions of the website.<br>CD4: https://www.abcam.com/cd4-antibody-panel-epr6855-sp35-bl-155-1c11-ab252199.html<br>CD8: https://www.abcam.com/cd8-alpha-antibody-sp16-ab101500.html<br>HSV: https://www.genetex.com/PDF/Download?catno=GTX73373<br>IDH1 R132H: https://www.dianova.com/en/shop/dia-h09-anti-idh1-r132h-hu-from-mouse-h09-unconj/<br>MGMT: https://www.abcam.com/mgmt-antibody-mt31-ab39253.html

Eukaryotic cell lines

Policy information about cell lines

Cell line source(s)	WHO Vero cells, Seed lot 10-87 (RIKEN, Ibaraki, Japan).
Authentication	Obtainment of WHO Vero cells from WHO was authenticated by RIKEN.
Mycoplasma contamination	Cells were tested negative for mycoplasma contamination.
Commonly misidentified lines (See ICLAC register)	N/A

Human research participants

Policy information about studies involving human research participants

Population characteristics	A total of 13 patients were included in the study who were diagnosed with recurrent or progressive glioblastoma. The median age (range) was 46 (35-76) years; 8 patients were male, and 5 patients were female; 5 patients were undergoing the first recurrence and 8 patients were undergoing the second recurrence. Other covariate-related characteristics of the 13 patients are included in Table 1.
Recruitment	Study participants were enrolled through patient referrals or the website of the Institute of Medical Science Hospital, the University of Tokyo. A meeting by the Eligibility Assessment Committee took place before every enrollment, and all eligible patients were enrolled sequentially without exception and intentional selection.
Ethics oversight	The study protocol was approved by the Ministry of Health, Labour and Welfare of Japan (MHLW) on May 11, 2009 for the University of Tokyo Hospital and on March 22, 2012 for the Institute of Medical Center Hospital. This study was also reviewed and approved by the Gene Therapy Clinical Research Review Board of the participating institutions.

Note that full information on the approval of the study protocol must also be provided in the manuscript.

Clinical data

Policy information about clinical studies

All manuscripts should comply with the ICMJEguidelines for publication of clinical research and a completedCONSORT checklist must be included with all submissions.

Clinical trial registration	UMIN-CTR Clinical Trial registry (UMIN000002661)
Study protocol	Full trial protocol is provided with the manuscript.
Data collection	The target population of the study consists of patients with recurrent or progressive glioblastoma treated at the University of Tokyo Hospital and the Institute of Medical Science Hospital of the University of Tokyo between November 2009 and November 2014.
Outcomes	The primary endpoint was safety of G47Δ, assessed by the type, frequency, and severity of adverse events.<br>Secondary endpoints included tumor response in the 90-day observation period after G47Δ administration evaluated using WHO-Response Evaluation Criteria based on MRI examination.<br>Further details are recorded in the main manuscript.

Magnetic resonance imaging

Experimental design

Design type	MRI with contrast enhancement performed according the protocol schedules.
Design specifications	MRI with contrast enhancement performed according the protocol schedules.
Behavioral performance measures	This is not fMRI study.

Acquisition

Imaging type(s)	Enhanced 3D-T1WI
Field strength	1.5 Tesla
Sequence & imaging parameters	Standard parameters of the radiology departments of the institutions.
Area of acquisition	Whole brain

Diffusion MRI ☐ Used ☒ Not used

Preprocessing

Preprocessing software	No preprocessing performed, MR images directly analyzed in our PACS.
Normalization	N/A
Normalization template	N/A
Noise and artifact removal	N/A
Volume censoring	N/A

Statistical modeling & inference

Model type and settings	No statistical modeling and inference performed.
Effect(s) tested	N/A

Specify type of analysis: ☐ Whole brain ☐ ROI-based ☐ Both

Statistic type for inference (See Eklund et al. 2016)	N/A
Correction	N/A

Models & analysis

n/a	Involved in the study
☒	☐ Functional and/or effective connectivity
☒	☐ Graph analysis
☒	☐ Multivariate modeling or predictive analysis
